# Enhancing Cascade Reaction Efficiency by Local pH Regulation for Integrated Anodic H_2_O_2_ Generation and Ammoximation

**DOI:** 10.1002/anie.202515867

**Published:** 2025-09-23

**Authors:** Lejing Li, Jian Zhang, Carla Santana Santos, Ridha Zerdoumi, Sabine Seisel, Shubhadeep Chandra, Wolfgang Schuhmann

**Affiliations:** ^1^ Analytical Chemistry – Center for Electrochemical Sciences (CES) Faculty of Chemistry and Biochemistry Ruhr University Bochum Universitätsstraße 150 D‐44780 Bochum Germany; ^2^ Institute for Materials Faculty of Mechanical Engineering Ruhr University Bochum Universitätsstraße 150 44801 Bochum Germany

**Keywords:** Ammonia oxidation, Anodic hydrogen peroxide, Hydroxylamine, Local pH, Oxime

## Abstract

Cascade reaction strategies integrating electrochemistry with chemical transformations offer routes for the synthesis of value‐added chemicals. However, the efficiencies of such integrated processes get compromised due to competitive electrochemical reactions and incompatibility between electrochemical and chemical transformations. We report an integrated electrochemical–chemical coupling of anodic H_2_O_2_ generation with ammoximation for oxime synthesis. An FTO/Sb_2_WO_6_ anode was designed and optimized to anodically produce H_2_O_2_ with a maximum Faradaic efficiency (FE) of 87%. H_2_O_2_ from the anode oxidizes NH_3_ to NH_2_OH, which subsequently reacts with cyclohexanone to yield cyclohexanone oxime with 99% selectivity and a maximum electron efficiency (EE) of 81%. Continuous and adjustable H_2_O_2_ input ensures synchronization with the ammonia oxidation reaction while minimizing over‐oxidation of the reaction intermediates. *Operando* scanning electrochemical microscopy (SECM) revealed local pH shifts caused by the proton‐coupled electron transfer and its effect on the FE of H_2_O_2_ synthesis and competing NH_3_ oxidation, providing mechanistic insights for optimizing the reaction microenvironment. By regulating the electrolyte composition to modulate the interfacial pH, side reactions were suppressed and H_2_O_2_ generation was promoted, thereby enhancing cascade selectivity. This work highlights local pH regulation as a tool to improve reaction compatibility and efficiency in cascade electrosynthesis.

## Graphical abstract

This work integrates anodic H_2_O_2_ generation with ammoximation for efficient oxime synthesis. By regulating the local pH through electrolyte engineering, the system achieves 81% electron efficiency (EE) and 98% H_2_O_2_ utilization, offering a generalizable strategy for cascade electrochemical–chemical processes.

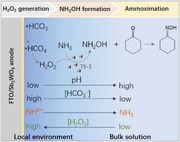

## Introduction

The synthesis of valuable precursors for important chemicals often involves complex reaction sequences.^[^ ^1^, ^2^ ^]^ In general, achieving the activation of multiple reactants and the formation of new chemical bonds in a single step remains challenging. Cascade reactions, which enable sequential chemical bond transformations toward final products, offer an intensified approach for synthesizing numerous precursors, particularly valuable ring molecules.^[^ ^3^, ^4^, ^5^ ^]^ These reactions can be achieved via spatial integration of active sites on a heterogeneous catalyst or by temporal coupling of electrochemical steps with chemical reactions in the bulk solution.^[^ ^6^, ^7^, ^8^, ^9^ ^]^ Integrating electrocatalysis with subsequent chemical conversions, or vice versa, has emerged as a promising route for synthesizing chemical commodities that are difficult to access via single‐step electrocatalysis or that rely heavily on stringent catalyst design or noble metals. For example, direct electrochemical oxidation of ethylene to ethylene oxide suffers from uncontrolled over‐oxidation and by‐product formation. Leow et al. addressed this by using Cl_2_ generated at the anode as a diffusional redox mediator, leading to hypochloride mediated partial oxidation with an overall Faradaic efficiency (FE) of ∼70%.^[^ ^10^ ^]^ Similarly, direct electrochemical generation of propylene oxide (PO) from propylene suffers from poor FE and dependence on Pd/Pt‐based catalysts.^[^ ^11^ ^]^ Chi et al. developed a cascade method involving electrolysis for bromine generation followed by chemical conversion of propylene to PO with an FE as high as 91%.^[^ ^12^ ^]^

**Scheme 1:**
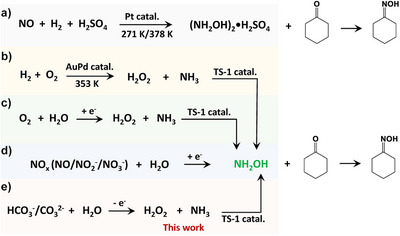
Strategies for cyclohexanone oxime synthesis by integrating thermocatalysis/electrocatalysis with chemical reactions. a) Cyclohexanone‐hydroxylamine method using hydroxylamine sulfate. b) Cyclohexanone ammoximation by in situ generated H_2_O_2_ from H_2_ and O_2_. c) Cyclohexanone ammoximation by in situ generated H_2_O_2_ from electrochemical O_2_ reduction. d) Electrosynthesis of NH_2_OH by NO_x_ reduction. e) Proposed anodic H_2_O_2_ generation and the integration with cyclohexanone ammoximation.

Cyclohexanone oxime is a crucial intermediate for producing caprolactam, a key monomer for nylon‐6 production. The global market for nylon‐6 is projected to reach USD 26040 million by 2032, expanding at a compound annual growth rate (CAGR) of 5.9%. Conventionally, cyclohexanone oxime is synthesized via the reaction of cyclohexanone with costly hydroxylamine sulfate under harsh conditions using explosive H_2_ and a noble metal catalyst at high temperatures, raising safety and cost concerns (Scheme 1a).^[^ ^13^ ^]^ Alternatively, hydroxylamine (NH_2_OH), the essential intermediate for cyclohexanone ammoximation, can be generated either by electrochemical reduction of NO_x_ species (NO, NO_2_^−^, and NO_3_^−^)^[^ ^5^, ^14^, ^15^, ^16^, ^17^ ^]^ or by oxidation of NH_3_ by means of H_2_O_2_.^[^ ^18^ ^]^ The NO_x_ reduction route (Scheme 1d) suffers from poor selectivity due to over‐reduction of NH_2_OH to NH_3_.^[^ ^15^, ^19^ ^]^ In contrast, oxidizing NH_3_ with the green oxidant H_2_O_2_ holds high potential for highly selective production of NH_2_OH (Scheme 1b,c).^[^ ^7^, ^18^ ^]^ Lewis et al. have demonstrated in situ H_2_O_2_ generation from H_2_ and O_2_ over titanium silicalite‐1 (TS‐1) supported Au‐Pd nanoparticles for cyclohexanone ammoximation (Scheme 1b).^[^ ^7^ ^]^

Anodic H_2_O_2_ generation offers a safe, green alternative that can be seamlessly integrated with ammoximation. In this approach, H_2_O_2_ produced at the anode oxidizes NH_3_ to NH_2_OH on TS‐1, which subsequently reacts with cyclohexanone to yield cyclohexanone oxime. The advantage lies in avoiding gaseous reactant handling, explosive oxidants, and toxic chemicals. Additionally, in situ H_2_O_2_ generation ensures a continuous and tunable H_2_O_2_ supply to match the ammonia oxidation rate while minimizing the over‐oxidation of the intermediate, NH_2_OH.

Nevertheless, integrating anodic H_2_O_2_ generation with the ammoximation reaction leads to unforeseen complications. On the one hand, the competitive oxygen evolution reaction (OER), shown in Equation (1) below, and the oxidation of NH_3_/NH_4_^+^ (Equations (2), (3), (4)) are thermodynamically more favorable than anodic H_2_O_2_ formation, which can limit the overall efficiency for H_2_O_2_/NH_2_OH generation. On the other hand, the compatibilities of the catalysts involved in cascade reactions and their respective reaction conditions are critical requirements for high overall efficiency of the integrated process. (Please refer to Equation 2, ref^[^ ^20^ ^]^; Equation 3 and 4, ref^[^ ^21^ ^]^ for further details)

(1) $$2\;H_{2}O⇌O_{2}+4\;H^{+}+4\;e^{-}\;E^{0}=1.23\;\text{V versus RHE}$$

(2) $$\begin{array}{cc} & {{\mathrm{NH}}_{4}}^{+}\left(\text{aq}\right)+2\;H_{2}O⇌{{\mathrm{NO}}_{2}}^{-}\left(\text{aq}\right)+8\;H^{+}+6\;e^{-}\\ & \;E^{0}=0.65\;\text{V versus SHE} \end{array}$$

(3) $$\begin{array}{cc} & 2\;NH_{3}\left(\text{aq}\right)⇌N_{2}\left(g\right)+6\;H^{+}+6\;e^{-}\;\\ & \;E^{0}=0.092\;\text{V versus RHE} \end{array}$$

(4) $$\begin{array}{cc} & 2\;NH_{3}+6\;OH^{-}⇌N_{2}\left(g\right)+6\;H_{2}O+6\;e^{-}\\ & \;E^{0}=0.092\;\text{V versus RHE} \end{array}$$

(5) $$2\;H_{2}O⇌H_{2}O_{2}+2\;H^{+}+2\;e^{-}\;E^{0}=1.76\;\text{V versus RHE}$$

Here, we report an integrated strategy for anodic H_2_O_2_ generation‐assisted cyclohexanone ammoximation, achieving ∼99% selectivity for cyclohexanone oxime and an overall electron efficiency (EE) of 81%. The FTO‐supported Sb_2_WO_6_ film was developed and optimized as the anode, providing a maximum FE of 87% for H_2_O_2_ generation in a KHCO_3_/K_2_CO_3_ mixture, while exhibiting low selectivity toward the oxidation of NH_3_/NH_4_^+^. The incompatibility between highly efficient H_2_O_2_ formation at the anode and subsequent ammoximation presents a key challenge in integrating these reactions into a cascade. This was addressed by electrolyte engineering guided by *operando* scanning electrochemical microscopy (SECM). SECM‐based local pH investigations revealed the H_2_O_2_ generation mechanism and were used to optimize the H_2_O_2_ generation rate. Moreover, oxidation of NH_3_/NH_4_^+^ was effectively suppressed by tuning the anolyte pH, the buffer capacity, and the NH_4_^+^ concentration. Furthermore, this strategy enabled efficient ammoximation of various ketones beyond cyclohexanone, demonstrating broad substrate applicability and providing a scalable platform for integrated green synthesis. This method avoids the high H_2_O_2_ concentration, transportation, and storage but still relies on H_2_ and expensive noble metals. This highlights the need for safer and more cost‐effective alternatives.

## Results and Discussion

### Anode Material Characterizations

**Figure 1:**
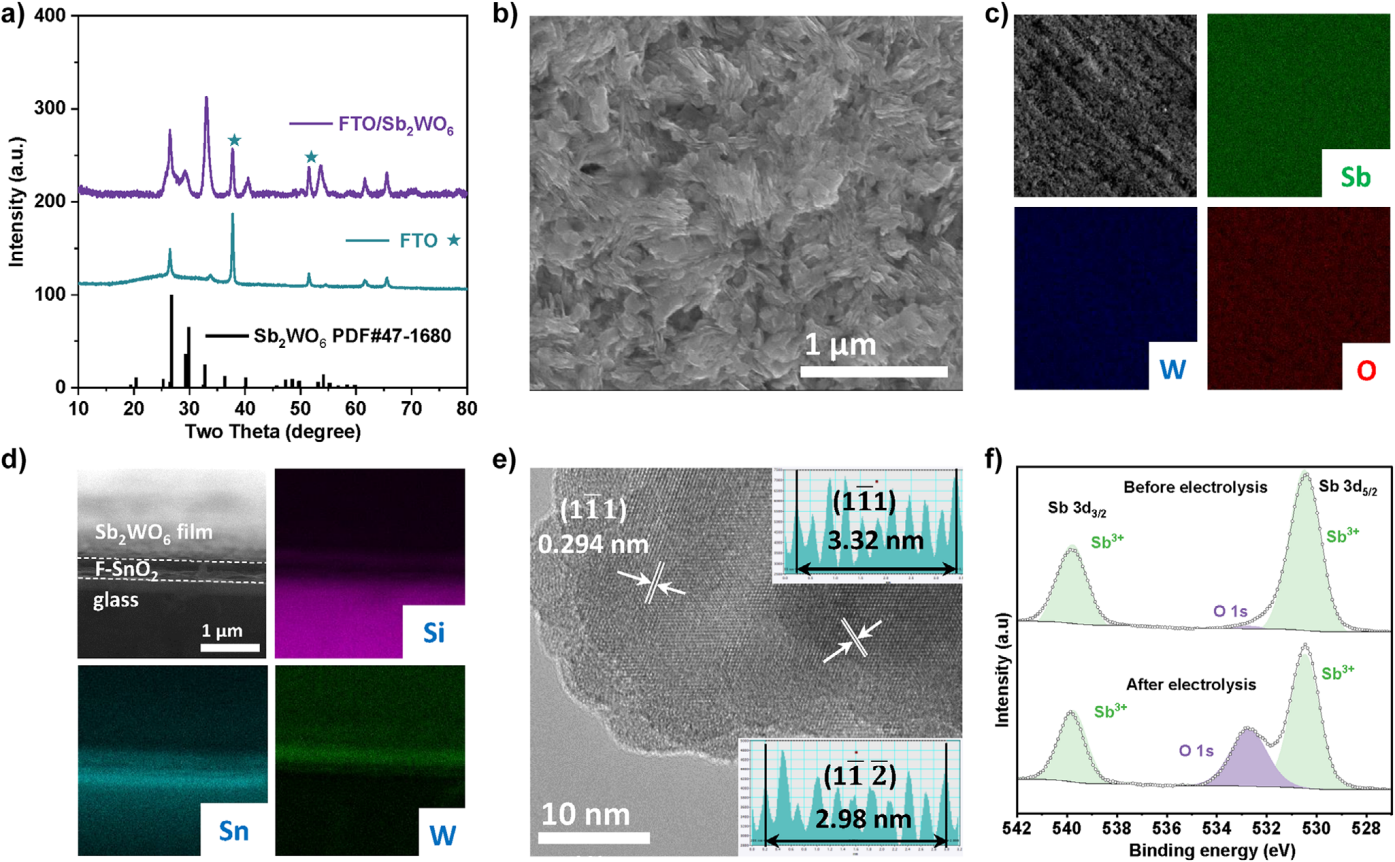
Catalyst characterization: a) The XRD pattern of the prepared FTO‐supported Sb_2_WO_6_ film (FTO/Sb_2_WO_6_). b) SEM images of the Sb_2_WO_6_ film. c) EDX images showing the distribution of Sb, W, and O on FTO. d) EDX images showing the distribution of Si, Sn, and W at the cross‐section of FTO/Sb_2_WO_6_. e) High‐resolution TEM image of Sb_2_WO_6_ scraped from FTO/Sb_2_WO_6_. f) High‐resolution XPS spectra of the Sb 3*d* region before and after electrolysis.

Sb_2_WO_6_ crystallizes in a layered Aurivillius‐type structure, consisting of perovskite‐like [WO_4_]^2−^ sheets alternately stacked with [Sb_2_O_2_]^2+^ layers. In this framework, W centers are tetrahedrally coordinated, and the [WO_4_]^2−^ layers in Sb_2_WO_6_ are reported to induce a more distorted lattice compared to other members of Aurivillius oxides.^[^ ^22^ ^]^ This suggests the presence of more unsaturated surface sites favorable for interfacial catalytic reactions. The basis for choosing Sb_2_WO_6_ can be found in the Experimental Section in the Supporting Information. The X‐ray diffraction (XRD) pattern of the catalyst film matches well with the reference pattern of Sb_2_WO_6_ (PDF#47–1680) (Figure 1a),^[^ ^22^ ^]^ confirming the successful synthesis of phase‐pure Sb_2_WO_6_ grown on FTO via a hydrothermal method. The SEM image (Figure 1b) reveals a clustered columnar morphology of the film. Energy‐dispersive X‐ray (EDX) mapping of the front side of the anode film confirms the homogeneous distribution of the W, Sb, and O (Figure 1c). Cross‐sectional SEM images clearly distinguish the catalyst layer and the underlying FTO substrate. The thickness of the Sb_2_WO_6_ layer is approximately 340 nm (Figure 1d). High‐resolution transmission electron microscopy (HRTEM) images (Figure 1e) show two sets of lattice fringes with inter‐planar spacing of 0.332 and 0.298 nm, which can be indexed to the (1$\bar{1}$1) and (1$\bar{1}\bar{2}$) facets of Sb_2_WO_6_, respectively. X‐ray photoelectron spectroscopy (XPS) was employed to analyze the valence states of the freshly prepared anode and the anode after performance evaluation (Figure 1f). The high‐resolution Sb 3*d* spectrum of the fresh sample can be deconvoluted into two main peaks at 539.8 and 530.5 eV, with a spin‐orbit splitting of 9.3 eV, corresponding to Sb^3+^ 3*d*_3/2_ and Sb^3+^ 3*d*_5/2_, respectively.^[^ ^23^, ^24^ ^]^ After electrolysis, the Sb 3*d* spectrum exhibits a negligible change in the Sb oxidation state. The additional peak at 532.7 eV in the O 1*s* region is attributed to adsorbed bicarbonate anions on the anode surface. The high‐resolution W 4*f* spectra (Figure S1) of both samples show the presence of mixed W^5+^ and W^6+^ valence states, with a W^6+^/W^5+^ ratio of approximately 6:4 in both cases, indicating that the valence state of W remains stable under electrolysis at high potentials. The stability of Sb_2_WO_6_ at harsh oxidation conditions was further evaluated by quantifying the concentration of leached metal(s) in the anolyte. Inductively coupled plasma mass spectrometry (ICP‐MS) analysis revealed that after 3 h of electrolysis at 2.83 V versus RHE, the concentration of leached Sb and W in the anolyte was at trace levels (around 300 ppb), indicating a good stability of the catalyst under operational conditions.

### Anodic H_2_O_2_ Generation Performance

**Figure 2:**
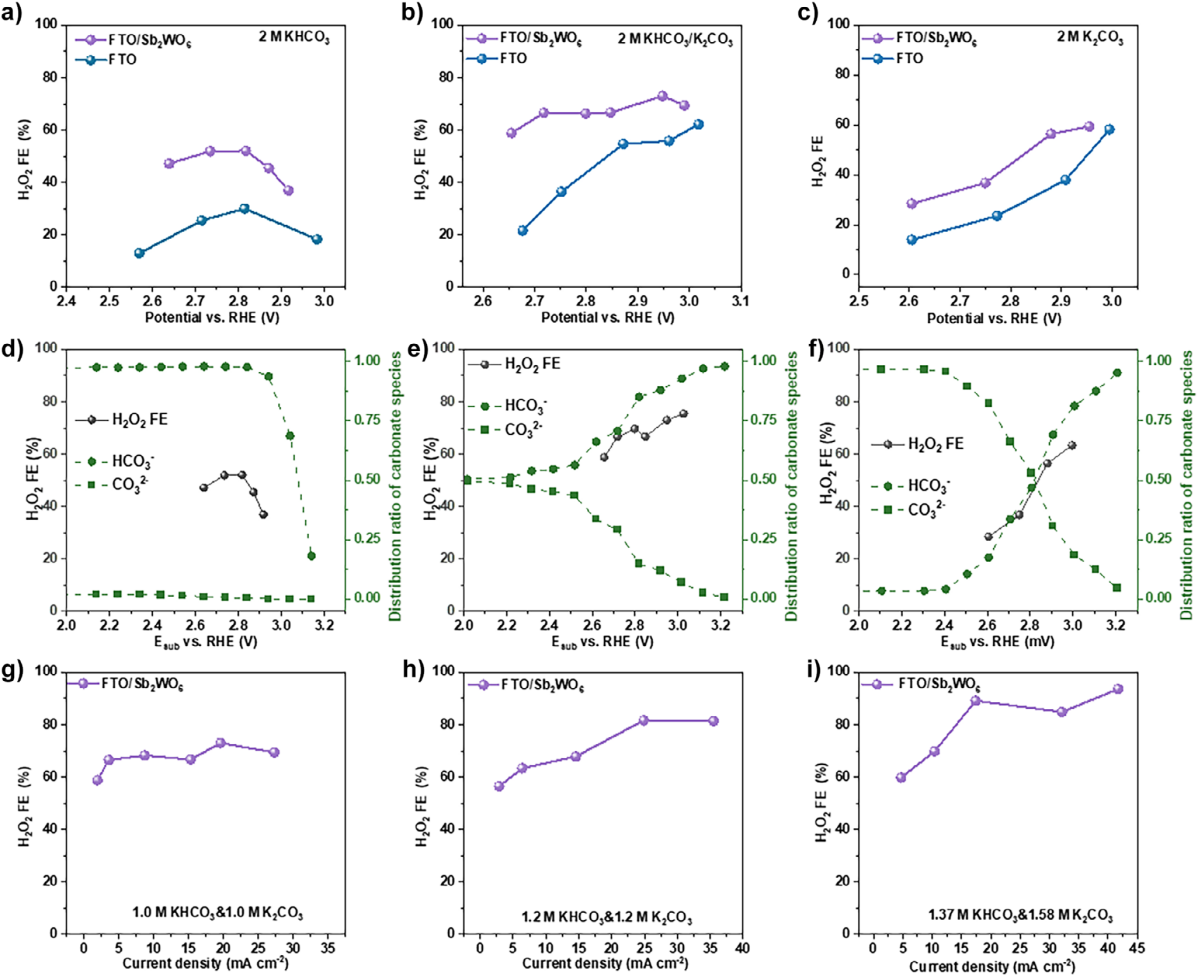
H_2_O_2_ FE of FTO/Sb_2_WO_6_ anode and local distribution of carbonate species. a)–c) Comparison of H_2_O_2_ FE of FTO and FTO/Sb_2_WO_6_ anodes in different anolytes. The FE toward H_2_O_2_ generation of FTO/Sb_2_WO_6_ anodes and *operando* changes in the equilibrium mole fractions of HCO_3_^−^/CO_3_^2−^ anions as a function of substrate potential (*E*_sub_) in d) 2.0 M KHCO_3_, e) a mixture of 2.0 M KHCO_3_ and 2.0 M K_2_CO_3_ with a pH value of 10, and f) 2.0 M K_2_CO_3_. The total concentration of carbonate‐based species for the three solutions is 2.0 M. g)–i) The H_2_O_2_ FE as a function of current density in carbonate‐based solution at pH 10 with different buffer capacity.

The pH influences multiple steps involved in cyclohexanone oxime production, including the H_2_O_2_ production rate, ammonia speciation, NH_2_OH reactivity, and ammoximation kinetics. To systematically investigate the pH effect, we first examined the H_2_O_2_ generation performance at FTO/Sb_2_WO_6_ anode. Previous studies have demonstrated that the anolyte composition critically influences the FE of anodic H_2_O_2_ generation,^[^ ^25^, ^26^, ^27^, ^28^, ^29^ ^]^ and the H_2_O_2_ formation may proceed through various reaction pathways, and intermediates, including hydroxyl radical, HCO_3_^−^, or CO_3_^2−^‐mediated mechanisms.^[^ ^25^, ^30^, ^31^, ^32^ ^]^ To eliminate the influence of competitive ammonia oxidation, we used KHCO_3_/K_2_CO_3_ mixtures as anolytes to elucidate the interplay between anolyte components and H_2_O_2_ generation efficiency at FTO/Sb_2_WO_6_ anode. The H_2_O_2_ generation performance of FTO/Sb_2_WO_6_ was evaluated in electrolytes containing the same total concentration of HCO_3_^−^/CO_3_^2−^ but at different pH values (Figure 2a–c). For comparison, the performance of bare FTO was also assessed as a reference. The results show that the FTO/Sb_2_WO_6_ anode consistently exhibited higher H_2_O_2_ FE than bare FTO in all tested anolytes, demonstrating the intrinsic high selectivity of the Sb_2_WO_6_ film toward H_2_O_2_ formation. Specifically, in 2.0 M KHCO_3_, the FTO/Sb_2_WO_6_ anode achieved an H_2_O_2_ FE of 40%–50% within the potential range of 2.50 to 2.90 V versus RHE. In a mixture of KHCO_3_/K_2_CO_3_ with a pH of 10 (Figure 2b), the anode exhibits even higher H_2_O_2_ FE, exceeding 60% over a broad potential range (2.65 to 3.10 V versus RHE). Switching to a pure K_2_CO_3_ anolyte (Figure 2c) did not result in a further increase in H_2_O_2_ FE. Instead, a steady increase was observed, with FE rising from 30% at 2.60 V to 60% at 2.92 V versus RHE. These results clearly demonstrate that carbonate‐based electrolytes at pH 10 promote H_2_O_2_ generation at FTO/Sb_2_WO_6_ anode.

### Investigation of Local pH Variations During H_2_O_2_ Generation

It is expected that the intrinsic layered structure of Sb_2_WO_6_, through its distinct surface terminations, along with the electrolyte composition and the turnover frequency of the electrochemical reactions, collectively regulate the local pH. In our experiments, we used the same catalyst to investigate the effects of electrolyte composition and applied potential on H_2_O_2_ selectivity. Therefore, the influence of the catalyst structure is not supposed to interfere with the absolute changes in local pH. During H_2_O_2_ generation, the oxidation of H_2_O, OH^−^, or HCO_3_^−^/CO_3_^2−^ species at the anode surface releases protons, resulting in a shift in the acid–base equilibrium in the anode microenvironment. Consequently, the distribution of anionic species in the vicinity of an operating anode deviates from that in the bulk anolyte. *Operando* local pH investigation provides critical insights into identifying the dominant species at the reaction interface under conditions favoring H_2_O_2_ formation at the FTO/Sb_2_WO_6_ anode and elucidating the associated reaction pathways. To this end, *operando* local pH measurements were performed using an Au microelectrode positioned in close proximity to an operating FTO/Sb_2_WO_6_ anode in carbonate‐buffered solutions of different pH values (for further details, see Experimental Section, Supporting Information). The local pH variation as a function of applied anode potentials, i.e., substrate potentials (*E*_sub_), was systematically studied in three anolytes.

As shown in Figure S2, a distinct anodic shift in the reduction peak potential of the Au tip (*E*(Au‐O_xRed_)) was observed for all cases as the anode potential was stepped from 1.5 to 3.0 V versus RHE, indicating progressive acidification within the anode microenvironment (for detailed descriptions, see Note S1). The decrease in local pH leads to the protonation of CO_3_^2−^ and HCO_3_^−^, forming HCO_3_^−^ and H_2_CO_3_, respectively, in accordance with the second (p*K*_a_ = 10.3) and first (p*K*_a_ = 6.3) dissociation constants of carbonic acid.

The molar fraction variations of carbonate species as a function of *E*_sub_ were calculated based on the local pH measurements. For each anolyte, the interfacial molar fractions of HCO_3_^−^ and CO_3_^2−^ were correlated with the H_2_O_2_ FE at varying *E*_sub_, as shown in Figure 2d–f. In 2.0 M KHCO_3_ solution (Figure 2d), the local molar fraction of HCO_3_^−^ starts decreasing at 2.83 V versus RHE, while the fraction of CO_3_^2−^ remains consistently low. Notably, the H_2_O_2_ FE reaches a maximum at 2.8 V versus RHE, after which it follows the decline trend of interfacial HCO_3_^−^ concentration, confirming a strong correlation between H_2_O_2_ FE and the availability of HCO_3_^−^ species at the anode surface as previously discussed.^[^ ^32^ ^]^ In a carbonate‐buffered anolyte at pH 10 (Figure 2e), the molar fraction of HCO_3_^−^ increases, while CO_3_^2−^ decreases over the potential range from 2.0 to 3.2 V versus RHE, indicating the progressive transformation of CO_3_^2−^ to HCO_3_^−^ at the reaction interface. Correspondingly, the H_2_O_2_ FE exhibits an increasing trend, rising from 58% at 2.65 V to 76% at 3.0 V versus RHE. In 2.0 M K_2_CO_3_ solution (Figure 2f), distinct changes in the molar fractions of HCO_3_^−^ and CO_3_^2−^ were observed, again reflecting the transformation of CO_3_^2−^ to HCO_3_^−^ at the interface. Correspondingly, the H_2_O_2_ FE increases, confirming the key role of HCO_3_^−^ in promoting H_2_O_2_ formation. The H_2_O_2_ FE at FTO/Sb_2_WO_6_ strongly depends on local HCO_3_^−^ concentration rather than CO_3_^2−^. Therefore, it is proposed that H_2_O_2_ formation on FTO/Sb_2_WO_6_ proceeds via a HCO_3_^−^‐mediated reaction pathway, in which HCO_3_^−^ is first oxidized to HCO_4_^−^, followed by its hydrolysis to yield H_2_O_2_.^[^ ^27^ ^]^

Based on the findings shown in Figure 2d–f, maintaining a high molar fraction of HCO_3_^−^ at the anode surface is critical for facilitating anodic H_2_O_2_ formation. To further improve H_2_O_2_ FE over a wide potential window, a pH 10 carbonate electrolyte was selected, and its buffer capacity was increased to mitigate the rapid depletion of local HCO_3_^−^ anions during electrolysis. As shown in Figure 2g–i, the H_2_O_2_ FE at the FTO/Sb_2_WO_6_ anode in pH 10 anolytes with varying carbonate concentrations was plotted against the current densities. It is found that increasing the total carbonate concentration from 2.0 M (Figure 2g) to 2.4 M (Figure 2i) leads to a significant enhancement in H_2_O_2_ FE. Further increasing the total carbonate concentration to 2.95 M (comprising 1.37 M HCO_3_^−^ and 1.58 M CO_3_^2−^) results in an additional improvement, achieving H_2_O_2_ FE of more than 80% over a current density range of 20 to 45 mA cm^−2^.

### Exploring NH_2_OH Production via NH_3_ Oxidation by H_2_O_2_

To evaluate hydroxylamine (NH_2_OH) production through NH_3_ oxidation using anodically in situ generated H_2_O_2_, NH_4_HCO_3_ was chosen to replace KHCO_3_ and serve as the nitrogen source. Cyclohexanone oxime formation was investigated in a custom‐designed H‐type cell (Figure S3) filled with an anolyte containing 10 mM cyclohexanone, first in the absence of the catalyst TS‐1 in four different anolytes containing varying NH_3_/NH_4_^+^ concentrations and different HCO_3_^−^/CO_3_^2−^ molar ratios (the exact cation and anion concentrations for anolytes 1–4 are listed in Table S1). Linear sweep voltammetry (LSV) recorded with and without cyclohexanone shows negligible differences, indicating that cyclohexanone is unlikely to undergo direct oxidation at the FTO/Sb_2_WO_6_ anode (Figure S4). After electrolysis for 60 min, the anolyte was analyzed by ^1^H nuclear magnetic resonance (^1^H NMR). The NMR spectra (Figure S5) exhibited no detectable signals corresponding to cyclohexanone oxime, indicating that the critical intermediate, NH_2_OH, was not generated in the four anolyte conditions. These findings suggest that neither direct NH_3_ oxidation at the FTO/Sb_2_WO_6_ anode nor the presence of H_2_O_2_ alone is sufficient for the generation of NH_2_OH and cyclohexanone oxime.

**Figure 3:**
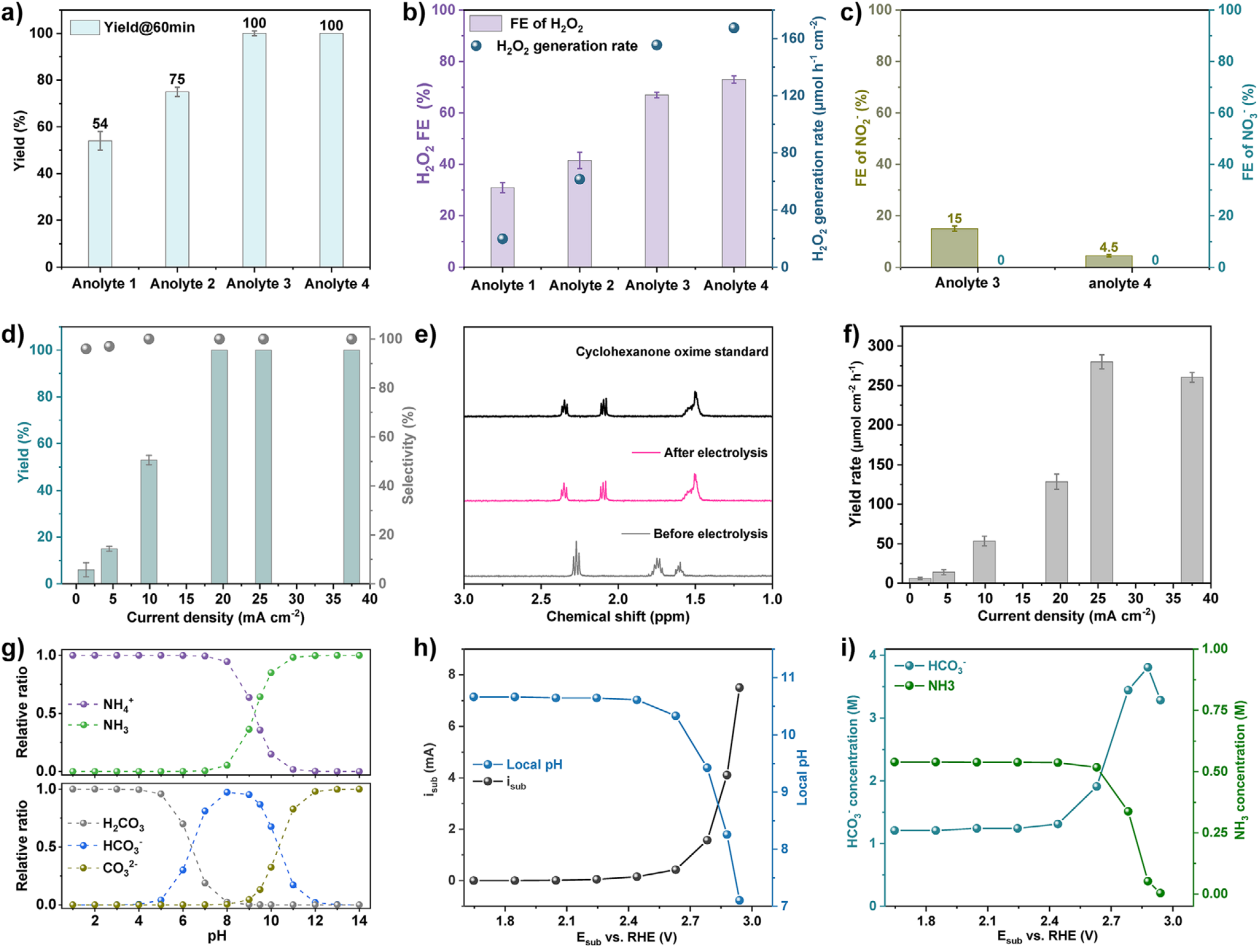
Cyclohexanone oxime synthesis in the anodic H_2_O_2_ system. a) Yield of cyclohexanone oxime after 60 min electrolysis at 2.78 V versus RHE in anolyte 1 to 4. The composition details of the electrolytes can be found in Table S1. b) FE and H_2_O_2_ generation rate of FTO/Sb_2_WO_6_ anodes at 2.78 V versus RHE in anolytes 1, 2, 3, and 4. c) FE of NO_2_^−^ and NO_3_^−^ at the FTO/Sb_2_WO_6_ anode at 2.78 V versus RHE in anolytes 3 and 4. d) Current density‐dependent cyclohexanone oxime yield and selectivity. e) ^1^H NMR of cyclohexanone oxime standard and the anolyte before and after electrolysis. f) Current density‐dependent cyclohexanone oxime yield rate in anolyte 4 using FTO/Sb_2_WO_6_ as the anode. g) The molar ratio of HCO_3_^−^/CO_3_^2−^/H_2_CO_3_ and NH_3_/NH_4_^+^ as a function of pH. h) Local pH changes as a function of anode potential in anolyte 4. i) The local concentrations of HCO_3_^−^ and NH_3_ as a function of the anode potential.

In subsequent experiments, TS‐1 was introduced into the anolyte to catalyze NH_3_ oxidation to NH_2_OH using anodically generated H_2_O_2_. As shown in Figure 3a, after electrolysis at 2.78 V versus RHE for 60 min, the oxime yield reaches 54% in anolyte 1 and 75% in anolyte 2, indicating that increasing the pH from 8.3 to 9.6 results in a 21% increase in oxime yield. Notably, the yield reaches 100% when the total HCO_3_^−^/CO_3_^2−^ concentration is increased from 2.0 M (anolytes 1 and 2) to above 3.3 M (anolytes 3 and 4). This improvement is attributed to the increased H_2_O_2_ generation rate in anolyte with higher buffer capacity, as discussed in Figure 2. Next, the selectivity of the oxidation reactions occurring at the FTO/Sb_2_WO_6_ anode was investigated (Figure 3b). The H_2_O_2_ FE reaches 31%, 41%, 67%, and 73% at 2.78 V versus RHE in anolytes 1, 2, 3, and 4, respectively. The missing FE is mainly due to the OER and the competing NH_3_/NH_4_^+^ oxidation, with the latter not only lowering the H_2_O_2_ selectivity but also reducing the availability of NH_3_ for the ammoximation reaction.

### Investigation of Ammonia Oxidation at the Anode

To further investigate direct ammonia oxidation at the anode, the FE for ammonia oxidation was examined using anolytes 3 and 4, as they exhibited a high FE of H_2_O_2_. As previously reported, NH_3_ tends to be oxidized to NO, NO_2_^−^, or NO_3_^−^ on transition metal‐based catalysts at high overpotentials.^[^ ^33^ ^]^ The amount of formed NO_2_^−^ and NO_3_^−^ was quantified by means of ion chromatography. After electrolysis at 2.78 V versus RHE, no NO_3_^−^ was detected in any of the anolytes (Figure 3c). When the NH_3_/NH_4_^+^ concentration decreases from 1.0 M (in anolyte 3) to 0.56 M (anolyte 4), the FE for NO_2_^−^ correspondingly decreases from 15% to 4.5%. The relatively low FE for NO_2_^−^ across the anolytes indicates that FTO/Sb_2_WO_6_ exhibits low activity toward NH_3_/NH_4_^+^ oxidation. The pH of the anolyte primarily governs the speciation of ammonia. NH_4_^+^ is generally considered electrochemically inert,^[^ ^34^ ^]^ whereas NH_3_ with the lone‐pair electrons is more readily adsorbed onto the catalyst surface at more alkaline conditions, thereby facilitating the NH_3_ oxidation reaction.

Based on these results, anolyte 4 was selected for subsequent studies on cyclohexanone oxime formation. After 60 min of electrolysis (Figure 3d), the oxime yield reaches 6%, 15%, and 53% at current densities of 1.4, 4.5, and 9.9 mA cm^−2^, respectively. Further increasing the current density leads to a complete conversion, achieving 100% oxime yield. As evidenced by the ^1^H NMR spectra, cyclohexanone oxime was identified as the sole product in the reaction system (Figures 3e and S6), demonstrating 100% product selectivity. Subsequently, the yield rate of cyclohexanone oxime was quantified (Figure 3f), and interestingly, a strong correlation was observed between the cyclohexanone oxime yield (Figure 3f) and the H_2_O_2_ generation rate (Figure S7), suggesting that the H_2_O_2_ production is the rate‐determining step in the overall oxime formation process.

### *Operando* Investigation of Local Anolyte Composition and its Impact on Anode Reactions

Changes in the local anolyte composition can significantly affect the H_2_O_2_ FE of FTO/Sb_2_WO_6_ anode, as revealed in Figure 2. Meanwhile, direct NH_3_ oxidation is highly pH‐sensitive. Therefore, *operando* monitoring of the local anolyte composition is essential for understanding the competitive reactions occurring at the anode. As shown in Figure 3g, the speciation of NH_3_/NH_4_^+^ and HCO_3_^−^/CO_3_^2−^ is highly pH‐dependent, indicating that their concentrations dynamically change during electrolysis. To evaluate these variations, *operando* measurements of local pH in the vicinity of FTO/Sb_2_WO_6_ anode were conducted in anolyte 4 (details provided in Figure S8). As the anode potential stepped to higher potentials, the increasing oxidation current caused a more pronounced pH decrease (Figure 3h), with a shift in local pH from 10.6 at 2.2 V to 8.2 at 2.85 V versus RHE. Correspondingly, the local concentrations of NH_3_ and HCO_3_^−^ were calculated, as shown in Figure 3i. It reveals that the local concentration of HCO_3_^−^ reaches its maximum near 2.85 V versus RHE, while the NH_3_ concentration decreases to less than 0.05 M. In parallel, the H_2_O_2_ FE reaches its maximum, attributed to the high interfacial coverage of HCO_3_^−^ and the conversion of NH_3_ to NH_4_^+^. The increasing acidification of the anode microenvironment suppresses NH_3_ oxidation since NH_4_^+^ cannot be directly oxidized.^[^ ^35^ ^]^ At neutral and acidic conditions, the hydration shell of NH_4_^+^ hinders its adsorption onto the catalyst surface, thereby reducing catalytic activity. Additionally, NH_4_^+^ oxidation requires prior deprotonation, which introduces an additional kinetic barrier, resulting in sluggish kinetics. Furthermore, the lower FE for NO_2_^−^ observed in anolyte 4 compared to anolyte 3 could be ascribed to the low local concentration of NH_3_.^[^ ^36^ ^]^ These findings collectively indicate that the inhibition of NH_3_ oxidation and the promotion of H_2_O_2_ generation can be achieved simultaneously through interfacial pH regulation, governed by bulk pH, NH_4_^+^ concentration, and buffer capacity of the anolyte.

### Efficiency Analysis of H_2_O_2_ Utilization for NH_2_OH Formation and Cyclohexanone Ammoximation

**Figure 4:**
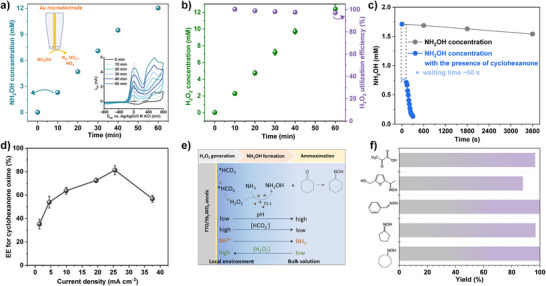
Overall EE and the applicability of the integrated system. a) Concentration of NH_2_OH in anolyte 4 as a function of electrolysis time. The inset shows the CVs recorded at the Au tip, indicating the increasing amount of NH_2_OH with electrolysis time. b) H_2_O_2_ concentration in anolyte 4 and the utilization efficiency of H_2_O_2_ for NH_2_OH formation. c) Time‐dependent NH_2_OH concentration in anolyte 4 in the absence or presence of 10 mM cyclohexanone. (a–c) FTO/Sb_2_WO_6_ anode polarized at 2.78 V versus RHE. d) EE for cyclohexanone oxime synthesis at different current densities. e) Schematic diagram for the coupled anodic H_2_O_2_ generation at the anode surface and the ammoximation reaction in the bulk solution. f) Synthesis of various ketone oximes through the anodic H_2_O_2_‐coupled cascade ammoximation.

Furthermore, the atom utilization efficiency of H_2_O_2_ for NH_2_OH formation was evaluated. The critical intermediate NH_2_OH was detected and quantified by its oxidation at an Au microelectrode owing to the high electrocatalytic activity of Au toward NH_2_OH oxidation.^[^ ^37^, ^38^ ^]^ Details of the calibration procedure are provided in Figures S9 and S10; Note S2. Next, the Au tip was dipped in anolyte 4. As shown in Figure 4a, the NH_2_OH concentration increases linearly as a function of electrolysis time. Meanwhile, the H_2_O_2_ concentration was also quantified (Figure 4b). Note that for H_2_O_2_ quantification, the analysis was performed in the absence of the TS‐1 catalyst. The H_2_O_2_ utilization efficiency for NH_2_OH formation reaches approximately 98% in the presence of TS‐1, indicating the excellent compatibility between anodic H_2_O_2_ generation and the subsequent oxidation of NH_3_ to NH_2_OH. The non‐catalytic reaction between NH_2_OH and cyclohexanone was investigated by monitoring the remaining NH_2_OH concentration in the presence of cyclohexanone by means of the Au tip response.

As shown in Figures S11 and 4c, NH_2_OH undergoes slow self‐decomposition in anolyte 4, with a 10% loss after 60 min at room temperature. In contrast, the addition of cyclohexanone led to rapid NH_2_OH consumption, indicating the high reactivity of NH_2_OH with cyclohexanone under the studied conditions. Kinetic analysis of the reaction between cyclohexanone and NH_2_OH revealed first‐order kinetics with respect to NH_2_OH concentration (Figure S12). This finding is consistent with the generally accepted reaction mechanism, wherein NH_2_OH acts as the nucleophile and attacks the electrophilic carbonyl carbon of cyclohexanone to form a transient intermediate, followed by dehydration to yield the corresponding oxime. Under the used conditions, the nucleophilic addition step is presumed to be the rate‐determining step, suggesting that the reaction rate is governed by the NH_2_OH concentration, which is ultimately determined by the H_2_O_2_ generation rate in the presence of a non‐limiting NH_3_ supply. The integrated system achieves a maximum EE of 81% for cyclohexanone oxime formation at 28 mA cm^−2^ current density, as shown in Figure 4d (details of the calculation are provided in the Supporting Information). This high EE is primarily attributed to the optimized electrolyte and the local acidification near the anode, which collectively suppress the OER and NH_3_ oxidation while simultaneously enhancing both formation and utilization of H_2_O_2_. The pH gradient across the diffusion layer was also investigated using the SECM‐positioned Au tip. Without forced convection, the diffusion layer thickness in anolyte 4 was derived to be about 3 mm (Figure S13), likely due to the high buffer capacity. The pH gradient across the diffusion layer from the anode surface to the bulk solution is shown in Figure S13, reflecting the concentration gradients of NH_3_ and HCO_3_^−^/CO_3_^2−^ species. We propose that the integrated system operates through spatial coupling of H_2_O_2_ generation at the anode surface, the oxidation of NH_3_ to NH_2_OH predominantly within the diffusion layer, and the subsequent ammoximation in the bulk solution. The high H_2_O_2_ utilization efficiency within the diffusion layer, where the pH remains in the optimal range of 9–10, is critical to the overall EE. Otherwise, at pH values exceeding 10, both H_2_O_2_ and NH_2_OH are susceptible to decomposition, reducing overall efficiency.^[^ ^26^ ^]^

Next, the durability of the FTO/Sb_2_WO_6_ anode was examined by performing consecutive cycling tests for the conversion of cyclohexanone to cyclohexanone oxime. As shown in Figure S14, the oxime yield remains above 90% throughout 10 consecutive cycles, confirming substantial stability of the Sb_2_WO_6_ film under operating conditions. The surface morphology and the LSV curves of the anode after the above cycles were examined. No obvious morphological changes were found compared to the as‐prepared Sb_2_WO_6_ film (Figures S15 and 1b), and the LSV curves (Figure S16) show only a slight decrease in the current density of the spent FTO/Sb_2_WO_6_ anode compared to the fresh one. To further assess the applicability of the H_2_O_2_ generation‐assisted ammoximation strategy, a variety of other substrates, including cyclooctanone, cyclopentanone, benzaldehyde, hydroxymethylfurfural, and pyruvic acid, was individually added to the anolyte. Remarkably, all the ketones and aldehydes were efficiently converted to their corresponding oximes with high yield (88%–100%) and excellent selectivity (>98%), as summarized in Figures 4f and S17.

## Conclusions

We have developed an integrated electrochemical–chemical strategy coupling anodic in situ H_2_O_2_ generation with TS‐1 catalyzed NH_2_OH formation, enabling direct ammoximation of cyclohexanone under mild conditions. An FTO/Sb_2_WO_6_ anode was developed and optimized, achieving a selectivity for H_2_O_2_ production as high as 87%. *Operando* SECM investigations revealed that regulating the NH_4_^+^ concentration, anolyte pH, and buffer capacity effectively suppresses the competing NH_3_ oxidation while enhancing H_2_O_2_ selectivity via microenvironment control. Guided by these insights, cyclohexanone oxime was produced with up to 100% yield and selectivity and a maximum EE of 81%. The system exhibits durability over ten consecutive cycles and broad applicability to various ketones and aldehydes, as confirmed by transforming various ketones and aldehydes into their corresponding oximes with high yields (88%–100%). This work not only provides new insights into the rational coupling of anodic H_2_O_2_ generation with fine chemical synthesis but also highlights the critical role of local pH regulation in maximizing cascade reaction efficiency by promoting target transformations and suppressing side reactions.

## Conflict of Interests

The authors declare no conflict of interest.

## Supporting information



Supporting Information

## Data Availability

All data associated with this article are available from the authors with reasonable request.
