# Supplementary material for: Enhancing Cascade Reaction Efficiency by Local pH Regulation for Integrated Anodic H2O2 Generation and Ammoximation
Source: Angew Chem Int Ed Engl. 2025 Sep 23;64(47):e202515867. doi: 10.1002/anie.202515867 (PMC12624321; doi:10.1002/anie.202515867)
Supplement: Supplementary file 1 — Supporting Information [file ANIE-64-e202515867-s001.docx]

Supporting Information

**Enhancing Cascade Reaction Efficiency by Local pH Regulation for Integrated Anodic H_2_O_2_ Generation and Ammoximation**

Lejing Li^1^, Jian Zhang^1^, Carla Santana Santos^1^, Ridha Zerdoumi^1,2^, Sabine Seisel^1^, Shubhadeep Chandra^1*^, Wolfgang Schuhmann^1^*

**1 Experimental section**

**1.1 Chemicals and materials**

SbCl_3_ (≥99.0%) and Na_2_WO_4_·2H_2_O (≥99.0%), fluorine doped tin oxide (FTO) with surface resistivity ~7 Ω/sq, deuterium oxide (99.9 atom% of D), maleic acid (≥99.0%), cyclohexanone (≥99.5%), hydroxylamine solution (50% wt% in H_2_O), cyclooctanon (≥99.0%), cyclopentaone (≥99.0%), benzaldehyde (≥99.0%), hydroxymethylfurfural (≥99.0%), pyruvic acid (≥99.0%), ethylene glycol (≥99.0%), and Nafion 117 membrane were purchased from Sigma-Aldrich. Titanium silicate molecule sieve (TS-1) was acquired from ACS materials.

**1.2 Basis for choosing Sb_2_WO_6_ as anode catalyst**

In this study, we choose Sb_2_WO_6_ as the anode catalyst because of its unique electronic structure and coordination environment of its constituent cations. First, we considered metal centers with electronically inert d^0^ or d^10^ configurations as potential active sites to avoid the strongly adsorbed oxygen intermediates (*OH, *O, *OOH) that are critical in the four-electron oxygen evolution reaction (OER) pathway. Previous DFT studies have shown that WO_3_ exhibits relatively weak adsorption of oxygen intermediates, and a low overpotential for H_2_O_2_ generation.^[1]^ This suggests that d^0^ W^6+^ centers in tetrahedral coordination lack the capacity to form strong metal-O bonds, thereby disfavoring OER. Moreover, as an acidic oxide, WO_3_ is known to suffer from electrochemical corrosion under alkaline conditions, whereas introducing Sb^3+^ to form the ternary oxide improves the stability of W coordination. Secondly, Sb-containing mixed metal oxides have been reported to exhibit high selectivity toward H_2_O_2_ generation.^[2]^ In Sb_2_WO_6_, the stereochemically active Sb^3+^ 5s^2^ lone pairs induce local lattice distortions, which elevate the valence band maximum via Sb-5s and O-2p orbital hybridization, thereby enhancing the oxidizing capacity toward bicarbonate activation. Finally, Sb_2_WO_6_ has been reported to exhibit high chemical stability under both acidic and alkaline conditions, ensuring good durability during electrochemical operation. ^[3]^

**1.3 Preparation of FTO/Sb_2_WO_6_**

In a typical procedure, SbCl_3_ (228 mg, 1 mmol) and Na_2_WO_4_·2 H_2_O (165 mg, 0.5 mmol) were separately dissolved in 10 mL of ethylene glycol (EG) under magnetic stirring at room temperature for 30 min. The two solutions were then mixed, followed by the addition of 10 mL deionized water and further stirring for 30 min. Next, the resulting mixture was transferred into a 50 mL Teflon-lined stainless steel autoclave. The FTO substrates (1 × 2 cm^2^) were sequentially cleaned with acetone and ethanol, dried in air and placed in the autoclave, leaning against the inner wall of the autoclave with the conductive side facing downward. The autoclave was sealed and heated at 160 °C for 10 h, then allowed to cool naturally to room temperature. The obtained catalyst film on FTO was subjected to calcination in air at 550 °C for 2 h with a heating rate of 3 °C min⁻¹.

**1.4 Catalyst characterizations and products quantification**

X-ray diffraction (XRD) patterns were recorded using a Bruker D8 Discover X-ray diffractometer equipped with a Cu Kα radiation source (λ = 1.5418 Å) over a two theta range of 10-90° with a step size of 0.02°. Scanning electron microscopy (SEM) images were acquired using a Quanta 3D FEG scanning electron microscope operated at 30.0 kV. Transmission electron microscopy (TEM) images were acquired on a JEOL microscope (JEM-2800) equipped with a Schottky-type emission source at 200 kV. X-ray photoelectron spectroscopy (XPS) was conducted with an AXIS Nova spectrometer (Kratos Analytical), equipped with a monochromatic Al Kα X‐ray source (1487 eV, 15 mA emission current) under a sample analysis chamber pressure of approximately 10^−8^ Torr. Photoelectrons were collected in the fixed transmission mode, while applying charge neutralization using an electron flood gun. A pass energy of 20 eV was used to acquire narrow spectra of the W 4*f*, Sb 3*d*, C 1*s*, and O 1*s* regions. The binding energies of the core-level spectra were calibrated based on the C 1s signal at 284.8 eV. Data processing and peak fitting of different components in the XPS spectra were conducted using the ESCApe software package (Kratos). Peak fitting was performed with a combination of Gaussian and Lorentzian line shapes, and the Shirley algorithm was used for background subtraction. Before inductively coupled plasma mass spectrometry (ICP-MS) measurements, a perchlorate precipitate was employed to significantly reduce the potassium content since high concentrations of alkali metal ions can suppress the detection sensitivity for other elements in ICP-MS analysis. Specifically, 1 mL of sample was mixed with 0.5 mL of 70% HClO_4_ and 0.3 mL of HNO_3_. The mixture was diluted to a final volume of 10 mL with 8.2 mL ultrapure water (resistivity: 0.055 μS cm^-1^), resulting in a 10% (v/v) solution. The above solution was cooled at 4 ^o^C for 90 min for facilitate KClO_4_ precipitation, followed by filtration using 0.2 μm syringe filters. Then the liquid sample was diluted to 0.01%, 0.1%, 1% and 10% with 2% nitric acid. ICP-MS determination was performed on an iCAP-RQ (Thermo Fisher), using argon plasma in collision cell mode (KED mode) with helium as collision gas. The concentration of Sb in the anolyte after the stability test was determined to be about 300 ppb. Nuclear magnetic resonance (NMR) spectroscopy was recorded on a Bruker 400 MHz NMR spectrometer. For NMR analysis, 400 μL of analyte was mixed with 100 μL of an internal standard (10 mM maleic acid in D_2_O). The H_2_O_2_ concentration was quantified by spectrophotometric determination of I_3_^-^.^[4]^ Prior to quantification, the aliquots were neutralized by adding sulfuric acid. The UV-vis absorption spectra were recorded on a Cary Series UV-Vis Spectrometer (Agilent Technologies). The concentrations of NO_3_^-^ and NO_2_^-^ produced via the oxidation of ammonia were quantitatively analyzed using ion chromatography (IC, 930 Compact IC Flex, Metrohm). The system was equipped with an anion exchange column (Metrosep A Supp 18 – 150/4.0) for the separation of anionic species and a conductivity detector (ProfIC Detector MF) for their detection. A. The IC system was operated with 23 mmol L^-1^ KOH solution used as the eluent (mobile phase) at a flow rate of 0.5 mL min⁻¹, and the column temperature was maintained at 30 °C. Calibration was performed using standard solutions of known nitrite and nitrate concentrations. Prior to analysis, all liquid samples were filtered through 0.2 µm syringe filters (Agilent Technologies) to remove particulates and ensure accurate quantification.

**1.5 Electrochemical measurements**

Electrochemical measurements were performed using a Gamry Reference 600 potentiostat/gal­vanostat in a divided glass H-cell separated by a Nafion 117 membrane. The H₂O₂ generation performances of FTO and FTO/Sb₂WO₆ electrodes were evaluated under potentiostatic conditions. Electrochemical impedance spectroscopy was conducted with an AC amplitude of 10 mV_pp_ over a frequency range of 1.0 to 100 kHz to determine the uncompensated solution resistance R_u_. After iR-drop compensation, the substrate potential was recalculated relative to the reversible hydrogen electrode (RHE) according to E_RHE_ = E_Ag/AgCl/3M KCl_ + 0.21 + 0.059 × pH – *i* × R_u_, and the pH values of the electrolytes were measured using a pH meter (FE28, Mettler Toledo).

**1.6 SECM-based local pH measurements**

Operando local pH measurements were conducted using a homemade SECM setup placed inside a Faraday cage. The entire setup was placed on an active damping table (Newport RS 2000) to avoid vibrational noise. Given that the molar fractions of HCO_3_^-^/CO_3_^2-^ and NH_3_/NH_4_^+^ are pH-dependent, real-time monitoring of local pH shift enables probing the distribution of critical anions and cations in the vicinity of the anode. The local pH changes at the operating FTO/Sb₂WO₆ anode were investigated by recording cyclic voltammograms (CVs) at a Au microelectrode (tip) positioned in close proximity to the anode biased at different potentials. The fundamentals of using Au tip as pH sensor can be found in our previous reports.^[5]^ The calibration curve of the Au tip response as a function of pH was obtained by recording the CVs at Au tip in a series of carbonate solutions with different pH (8.8, 9.7, 10.6, 12.7) and in a phosphate buffer with pH of 7.0. For the *operando* local pH measurements, Au tip with a diameter of 10 µm was first positioned in close proximity to the FTO/Sb₂WO₆ anode by employing the negative feedback approach method of SECM, based on the diffusion-limited O_2_ reduction reaction (ORR) in O_2_-saturated carbonate solutions. The Au tip was biased at 0.1 V vs. RHE to reduce O_2_. As Au tip gradually approached the substrate, the obstruction of O_2_ diffusion by the anode caused a declining ORR current at the Au tip. The approach was terminated once the ORR current reached a minimum, establishing a working distance of approxi­mately 10 µm. At this working distance, CVs were registered at the Au tip for the *operando* local pH measurements.

**1.7 Electron efficiency (EE) calculation of the tandem anodic H_2_O_2_ generation and ammoximation**

The overall efficiency of the tandem system depends on the efficiency of each step, *i.e.* the anodic H_2_O_2_ generation, oxidation of NH_3_ to NH_2_OH and the following reaction between NH_2_OH and cyclohexanone. We define the concept of utilization efficiency of H_2_O_2_ (UE_H2O2_) which represents the contribution of H_2_O_2_ to the formation of the key intermediate NH_2_OH, and also define the utilization of NH_2_OH (UE_NH2OH_)

$\mathrm{UE}_{H2O2}$ = $\frac{Z \times n_{NH2OH}}{n_{anode-H2O2}}$ ${\times100\%}$

Where *Z* represents the amount of H_2_O_2_ used for the production of hydroxylamine per unit, $n_{NH2OH}$ represents the amount of formed hydroxylamine, $n_{anode-H2O2}$ represents the total amount of H_2_O_2_ produced by the anode.

$\mathrm{UE}_{NH2OH}$ = $\frac{Z \times n_{oxime}}{n_{NH2OH}}$ ${\times100\%}$

here *Z* is equal to 1, $n_{oxime}$ represents the amount of the final product cyclohexanone oxime. But UE_NH2OH_ can get compromised if NH_2_OH decomposes in the reaction suspension.

Then the electron efficiency (EE) of the above tandem reaction can be expressed as:

$\mathrm{EE}_{oxime}$ = $\mathrm{FE}_{H2O2}{\times\mathrm{UE}}_{H2O2}$ ${\times\mathrm{UE}_{NH2OH}}$

**1.8 Calculations of the local species concentration**

According to the equilibrium for dissolved carbonate species in water, the dissociation of carbonic acid can be described by the following equilibrium equations:

H_2_CO_3_ ⇌ H^+^ + HCO_3_^-^  Eq. S1

$K_{1}$ = $\frac{\left[ H^{+} \right][{HCO}_{3}^{-}]}{[H_{2}CO_{3}]}$ = ${4.45\times10}^{-7}(25℃)$

HCO_3_^-^ ⇌ H^+^ + CO_3_^2-^  Eq. S2

$K_{2}$ = $\frac{\left[ H^{+} \right][{CO}_{3}^{2-}]}{[{HCO}_{3}^{-}]}$ = ${4.69\times10}^{-11}(25℃)$

since *K*_1_ and *K*_2_ are temperature-dependent constants, the distribution of carbonate species at a constant temperature is determined by the proton concentration in the solutions. The concentrations of HCO_3_^-^ and CO_3_^2-^ can be expressed as:

[HCO_3_^-^] = C_t_ × α_1_ Eq. S3

[CO_3_^2-^] = C_t_ × α_2_  Eq. S4

where C_t_ is the total amount of the carbonate concentration. α_1_ and α_2_ represent the distribution ratio of HCO_3_^-^ and CO_3_^2-^. These coefficients α_1_ and α_2_ can be expressed as a function of the proton concentration as follows:

$\alpha_{1}$ = $\frac{K_{1}\times\left[ H^{+} \right]}{\left[ H^{+} \right]\times\left[ H^{+} \right]+K_{1}\times\left[ H^{+} \right]+K_{1}\times K_{2}}$ Eq. S5

$\alpha_{2}$ = $\frac{K_{1}\times K_{2}}{\left[ H^{+} \right]\times\left[ H^{+} \right]+K_{1}\times\left[ H^{+} \right]+K_{1}\times K_{2}}$ Eq. S6

Thus, at a given temperature, the molar distribution of carbonate species in the solution can be calculated according to Eq. S5 and S6 using the known pH values.

Similarly, the dissociation of ammonia species can be described by:

NH_4_^+^ ⇌ H^+^ + NH_3_ Eq. S7

$K_{a}$ = $\frac{\left[ H^{+} \right]\left[ {NH}_{3} \right]}{\left[ {NH}_{4}^{+} \right]}$ = ${5.62\times10}^{-10}(25℃)$

At a constant temperature, the NH_3_ and NH_4_^+^ species should be distributed in a fixed proportion determined only by the proton concentration in the solutions.

${NH}_{3}$ = $\frac{K_{a}}{\left[ H^{+} \right] + K_{a}}$ Eq. S8

**2 Supplementary Figures**


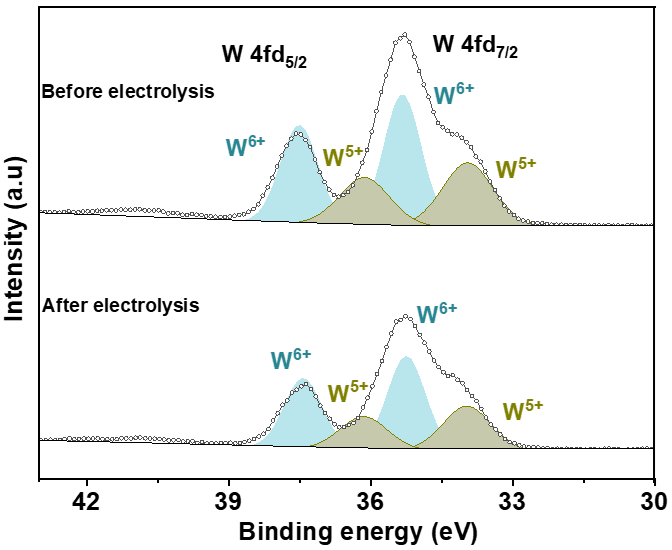


**Figure S1**. High resolution XPS spectra of the W 4*f* region before and after electrolysis.

**
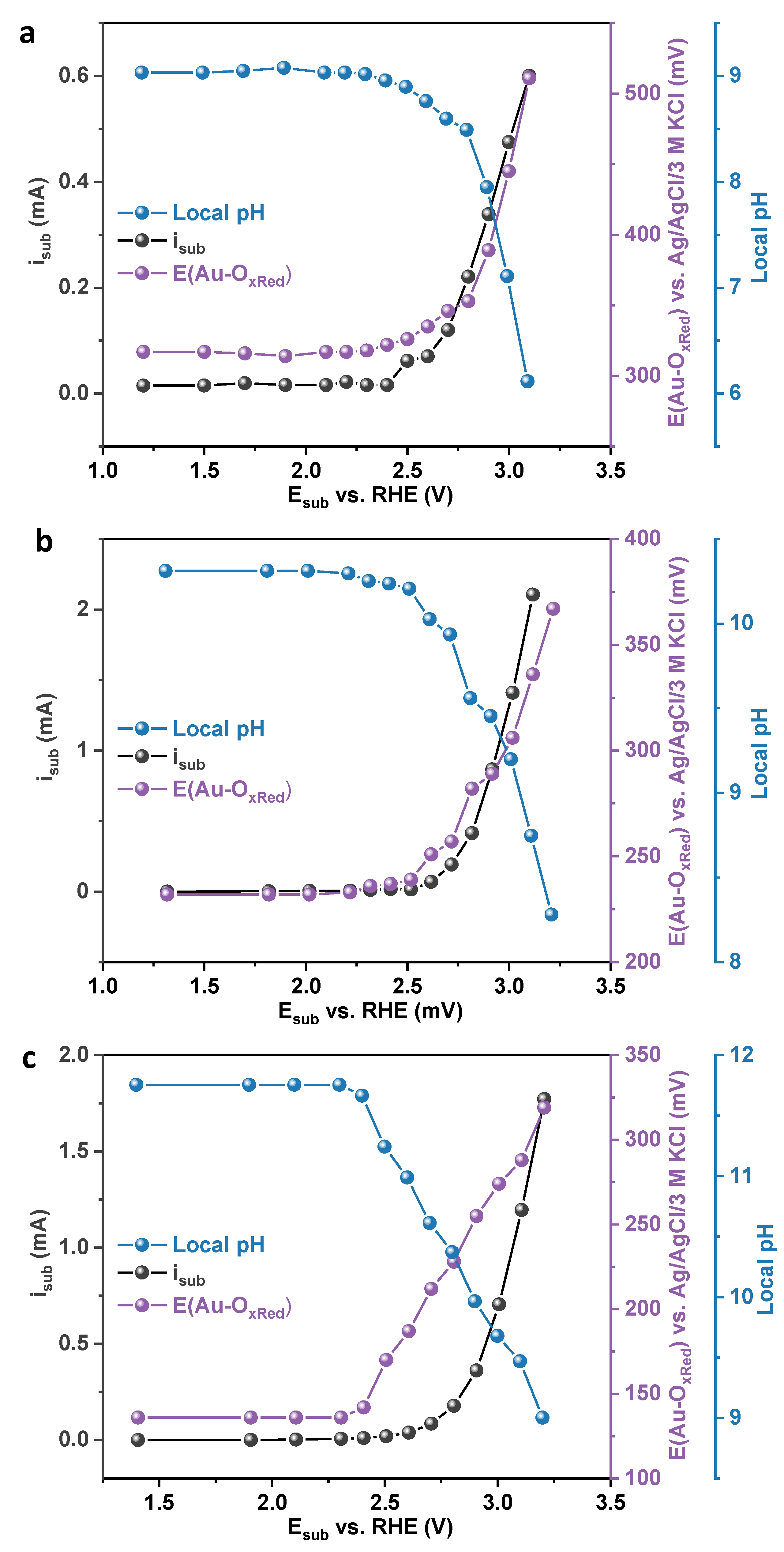
**

**Figure S2**. Extracted values of the oxidation current of FTO/Sb_2_WO_6_ anode, the reduction peak potentials of the Au tip (E(Au-O_Red_)), and the calculated local pH values as a function of anode potentials (E_sub_) in carbonate-based solutions with pH of (a) 9.0 (b) 10.3 and (c) 11.8.

**Supplementary Note 1:**

In 2 M KHCO_3_ (**Figure S2**a), when E_sub_ stepped from 1.2 V (no water oxidation) to 2.1 V vs. RHE, no significant increase in oxidation current is observed, and the local pH remains nearly unchanged. However, when E_sub_ stepped from 2.1 to 3.1 V vs. RHE, a sharp local pH drop from 8.6 to 6.0 was recorded, suggesting significant acidification and depletion of HCO_3_^-^ near the anode interface. In pH 10 anolyte (**Figure S2**b), an anodic peak shift of E(Au-O_xRed_) from 230 mV to 367 mV vs. Ag/AgCl/3 M KCl was observed when E_sub_ increased from 2.0 to 3.2 V vs. RHE, corresponding to a local pH decrease from 10.3 to 8.3. In 2 M K_2_CO_3_ (**Figure S2**c), a similar anodic shift in E(Au-O_xRed_) was recorded from 137 to 320 mV vs. Ag/AgCl/3 M KCl when E_sub_ stepped from 2.3 to 3.2 V vs. RHE, corresponding to a local pH decrease from 11.8 to 9.0.


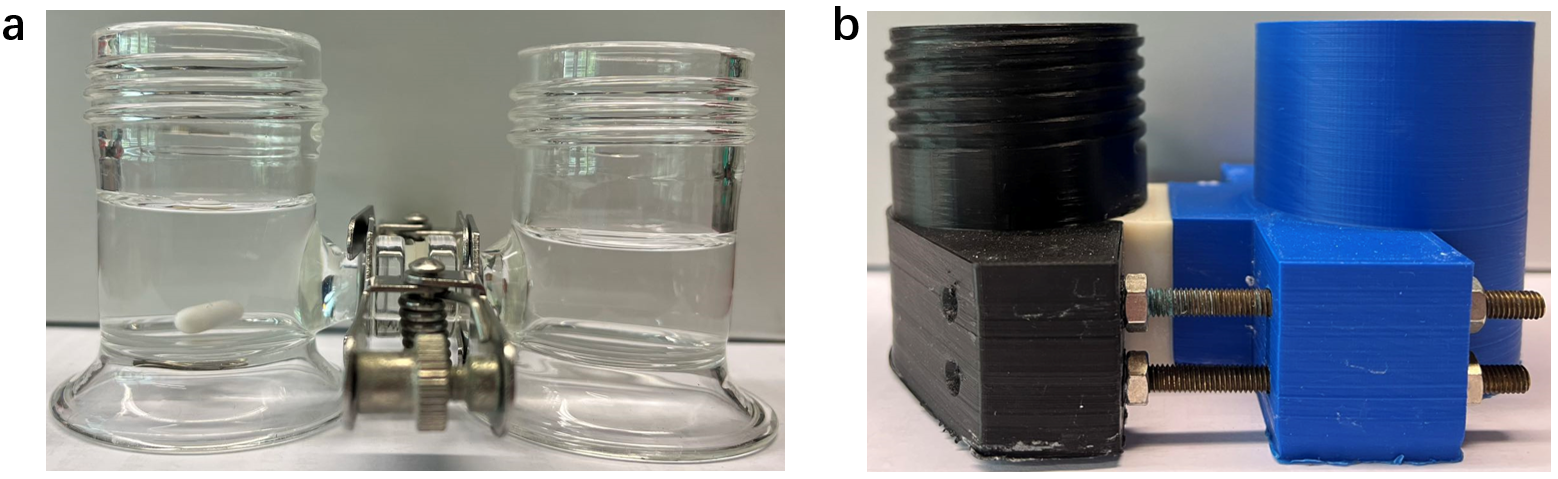


**Figure S3**. (a) The typical glass H-cell for H_2_O_2_ performance test. The bridge connecting the anolyte and catholyte compartments tends to cause TS-1 powder to accumulate in the corner of the cell, leading to poor dispersion. (b) Designed 3D-printed H-type cell for cascade oxime formation. The anolyte compartment has a cylindrical structure with an open window for membrane fixation, which improves the powder dispersion and facilitates the cascade reaction operation. The material for the anolyte compartment is made of polypropylene.

**
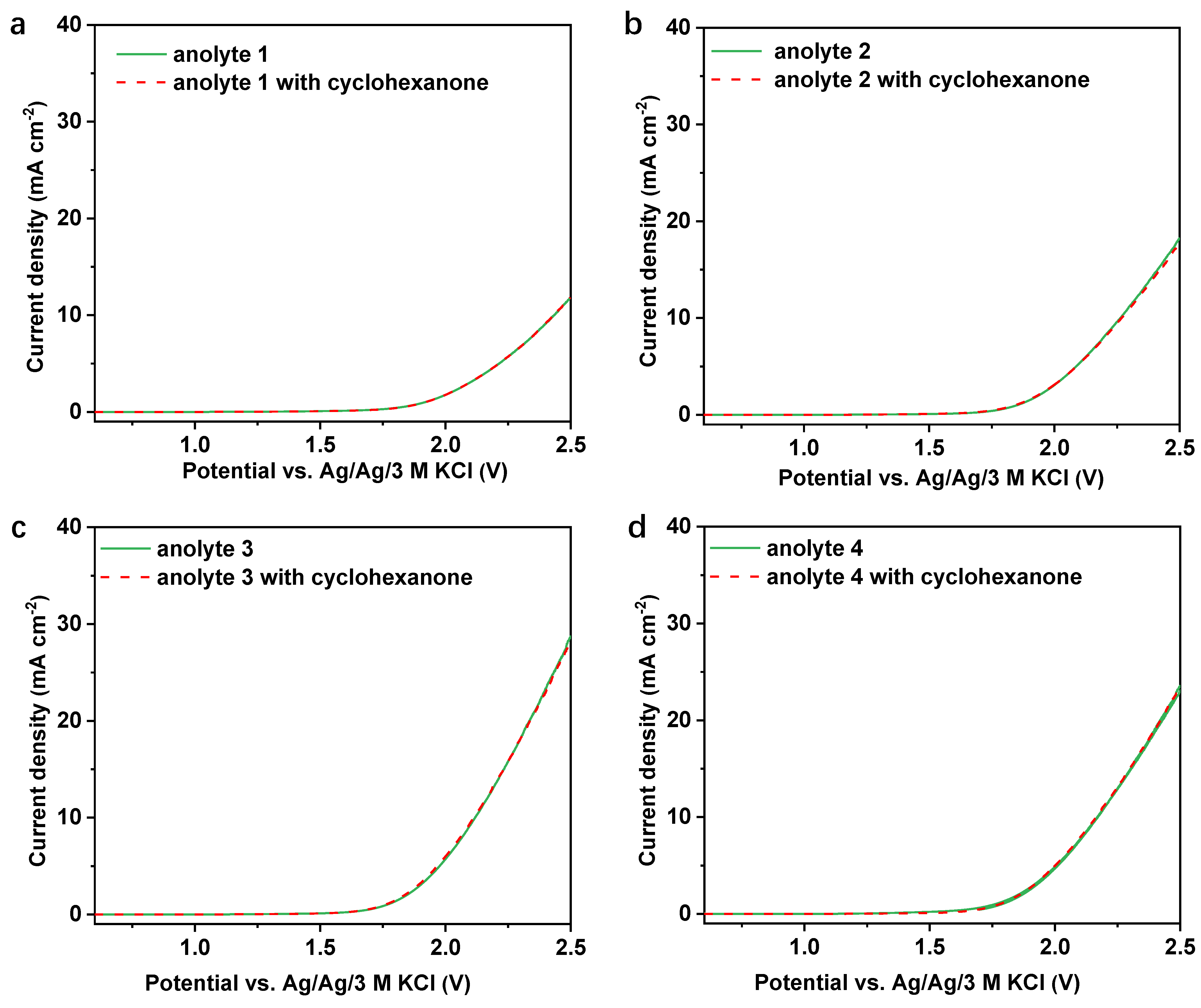
**

**Figure S4**. LSVs of an FTO/Sb_2_WO_6_ anode in anolytes 1, 2, 3 and 4 with and without cyclo­hexanone (10 mM). Measurements performed in the absence of TS-1. Compositions of anolytes 1-4 are presented in the **Table S1**.


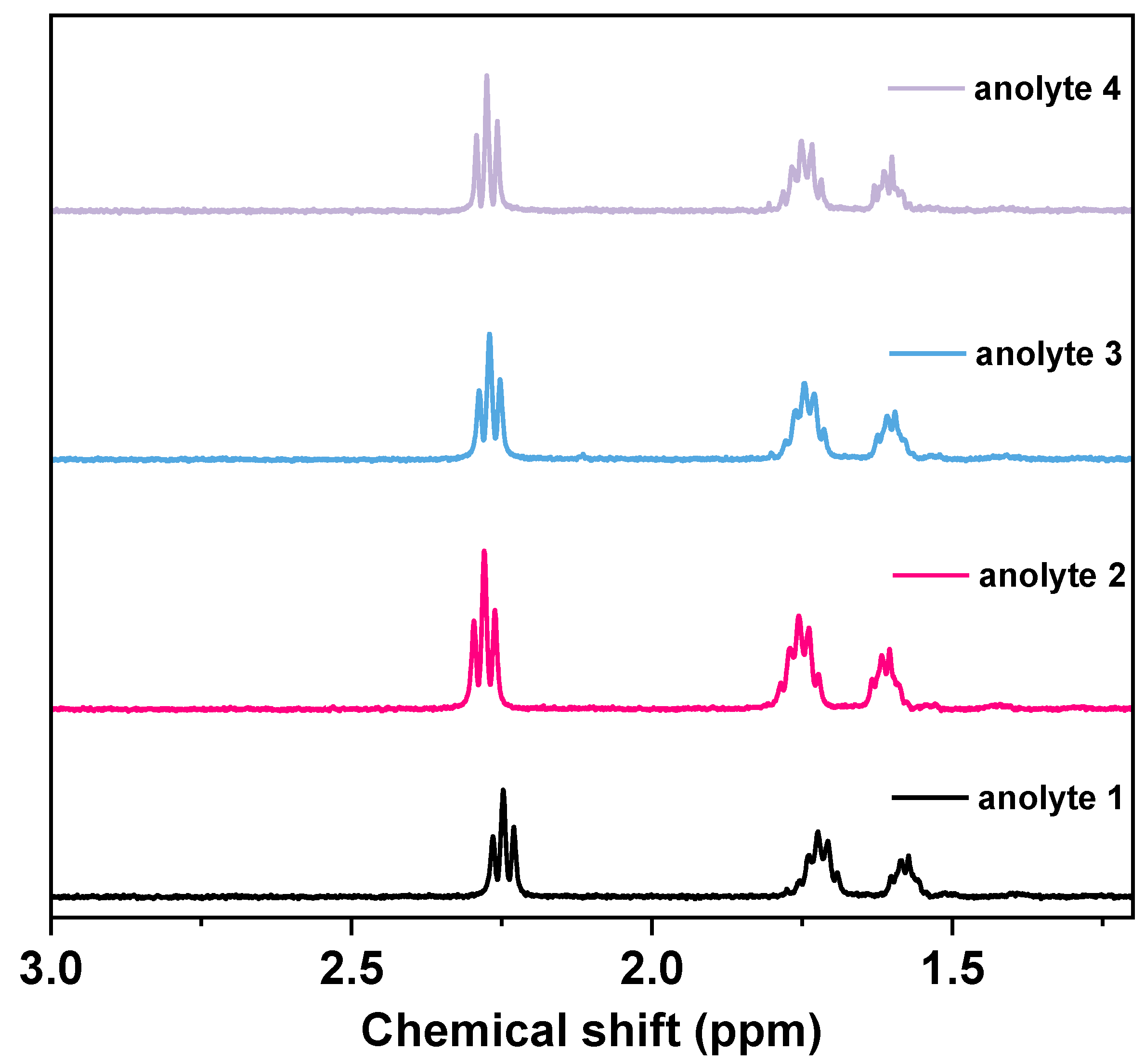


**Figure S5**. ^1^H NMR spectra of anolyte 1, 2, 3 and 4 after electrolysis at 2.78 V vs. RHE for 60 min in the absence of TS-1. The initial concentration of cyclohexanone was 10 mM in all anolytes.

**Table S1 Composition of anolytes 1, 2, 3 and 4**

| Anolyte | pH | NH_4_^+^ (M) | HCO_3_^-^/CO_3_^2-^ (M) | 2 M NH_4_HCO_3_ | 2 M KHCO_3_ | 2 M K_2_CO_3_ | 5 M K_2_CO_3_ |
| --- | --- | --- | --- | --- | --- | --- | --- |
| 1 | 8.3 | 1 | 2 | 10 mL | 10 mL |  |  |
| 2 | 9.6 | 1 | 2 | 10 mL |  | 10 mL |  |
| 3 | 10.9 | 1 | 3.5 | 10 mL |  |  | 10 mL |
| 4 | 10.6 | 0.56 | 3.33 | 5 mL | 5 mL |  | 8 mL |


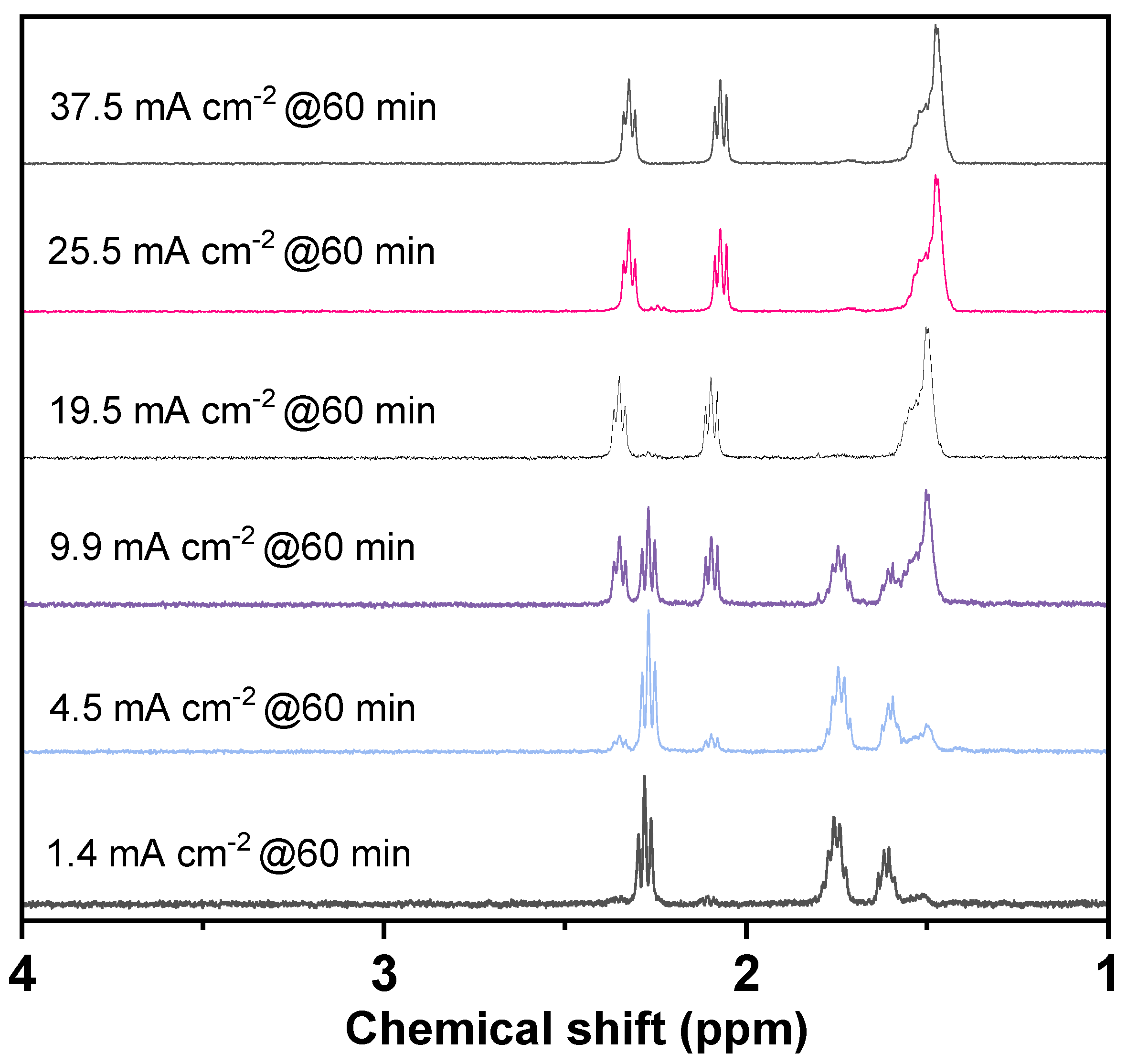


**Figure S6**. ^1^H NMR spectra of anolytes after 60 min-electrolysis at different current densities. At current densities equal and higher than 19.5 mA cm^-2^, the cyclohexanone oxime was identified as the sole product.

**
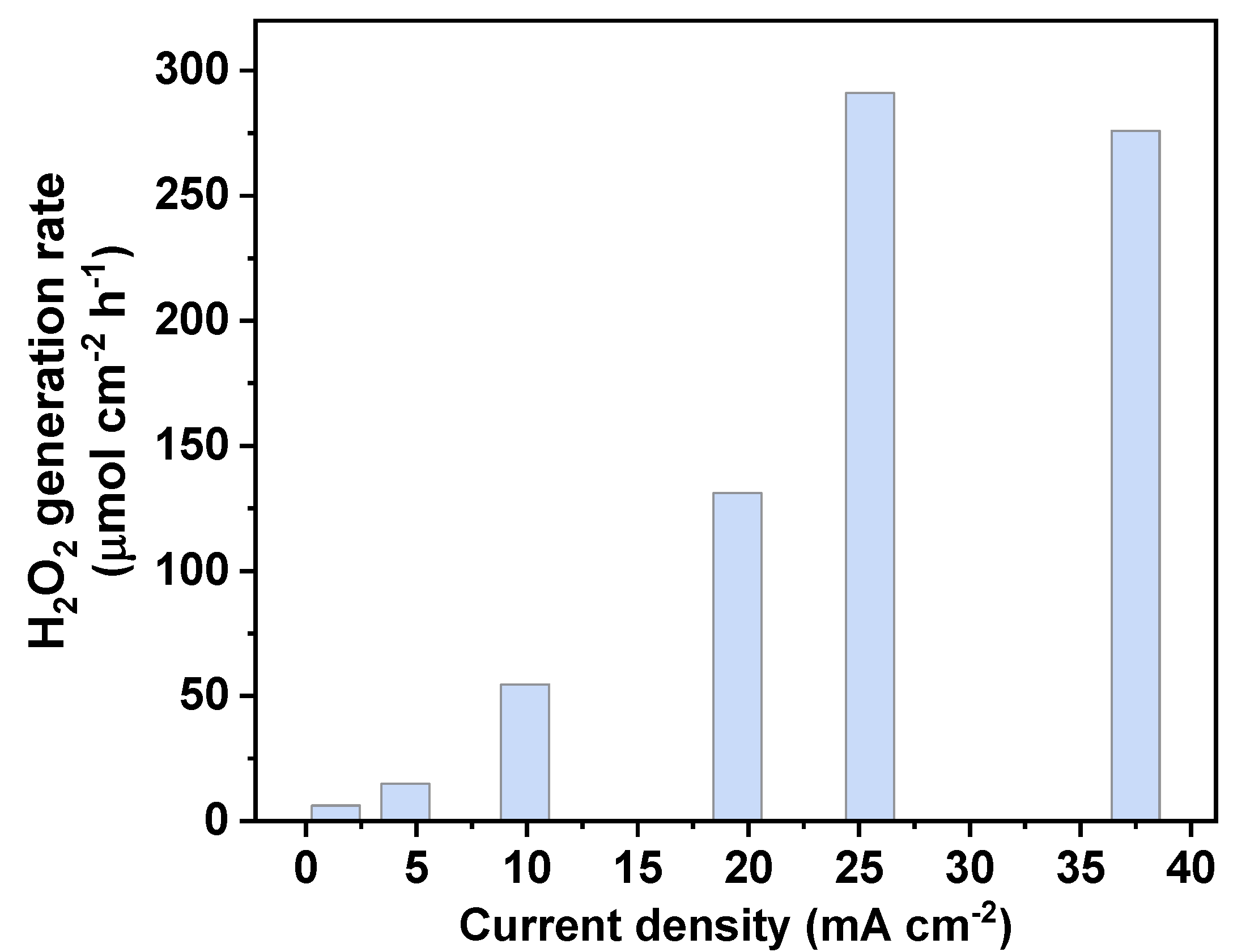
**

**Figure S7**. H_2_O_2_ generation rate at an FTO/Sb_2_WO_6_ anode in anolyte 4 at different current densities.


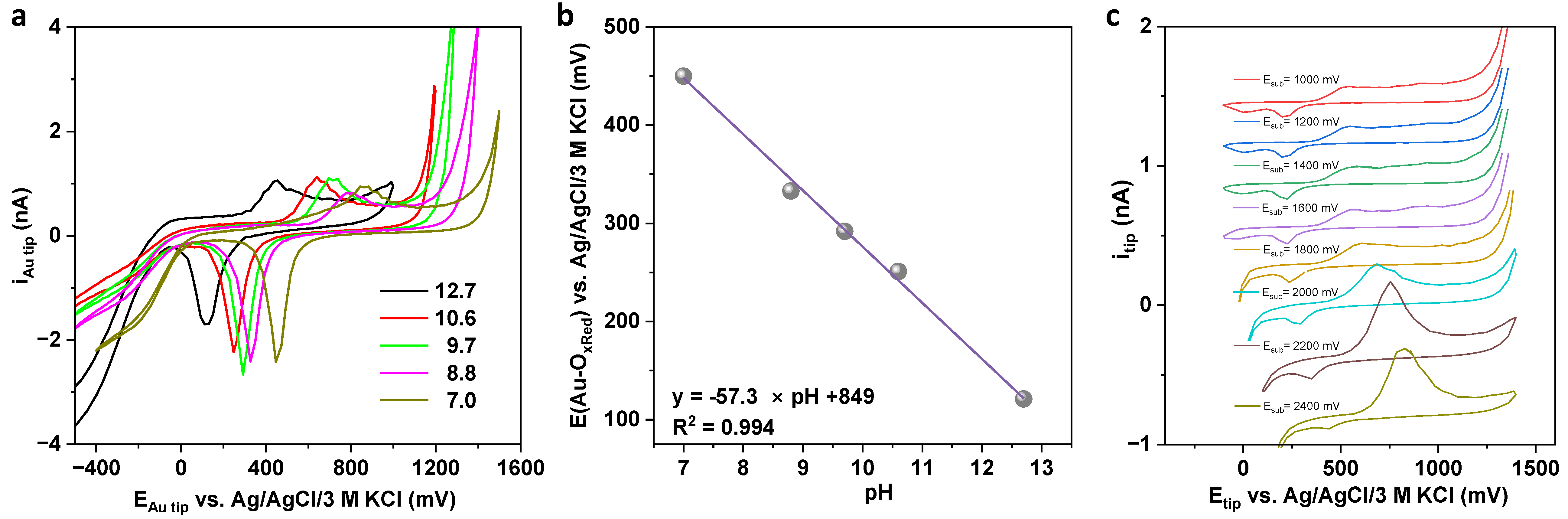


**Figure. S8**. (a) CVs at the Au tip in a series of calibration solutions with pH values ranging from 7.0 to 12.7. (b) Calibration curves derived from the reduction peak potentials of Au-O_x_ in Figure S8 (a) as a function of pH values. The reduction peak potentials were averaged over three measurements. The calibration curve in Figure S8b shows a slope of 57 mV pH^-1^. (c) CVs recorded at the Au tip in close proximity to the FTO/Sb_2_WO_6_ anode when a different potentials were applied to the FTO/Sb_2_WO_6_ anode in anolyte 4.


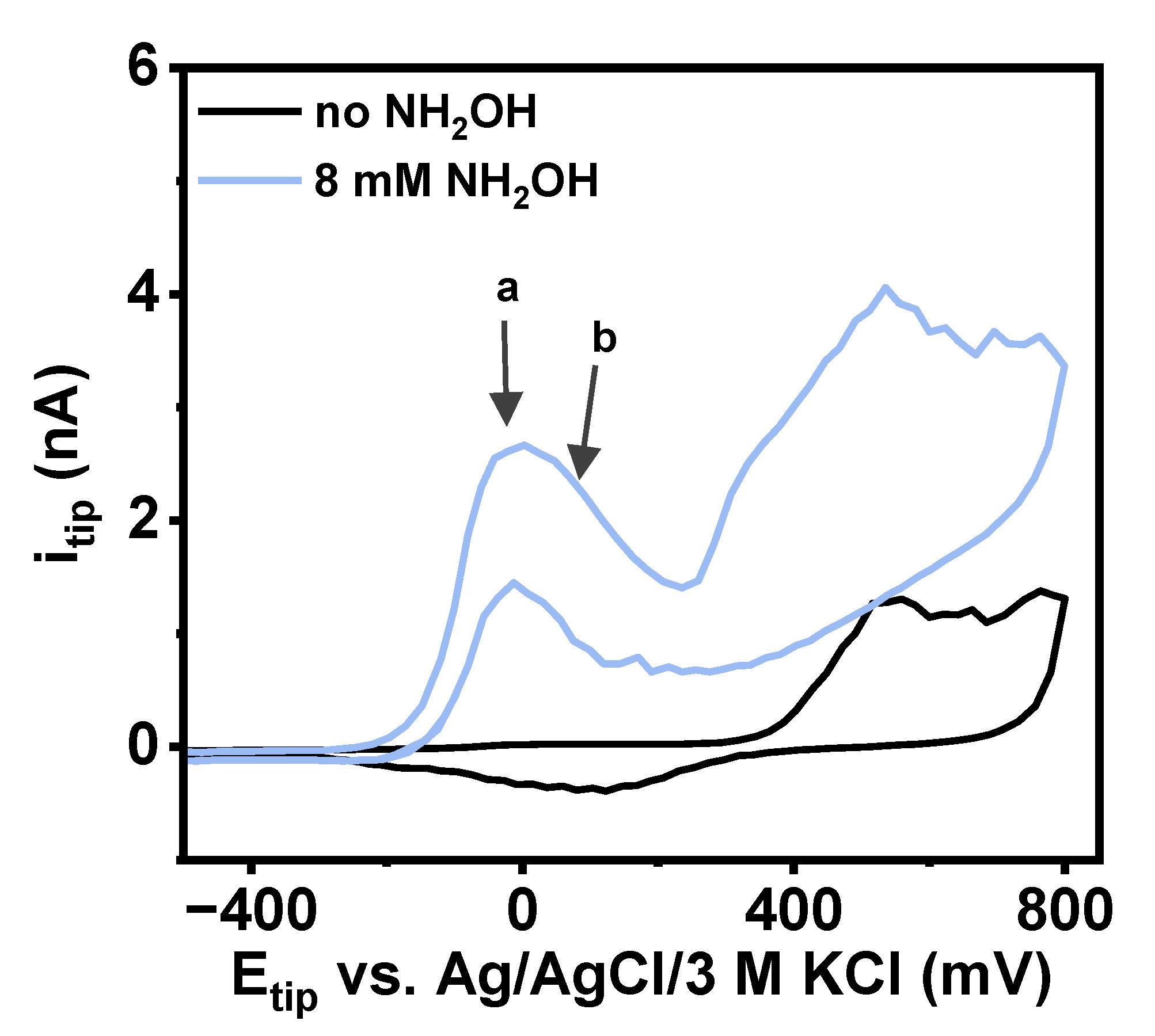


**Figure S9.** CVs at the Au microelectrode (25 μm diameter) in anolyte 4 with (blue curve) and without (black curve) hydroxylamine.

**Supplementary Note 2: The oxidation of NH_2_OH on Au microelectrode**

The oxidation of NH_2_OH is highly dependent on the solution pH. NH_2_OH presents two forms, the non-protonated form NH_2_OH and protonated form NH_3_OH^+^. The latter one has been assumed to be less active. It has been demonstrated that the oxidation of hydroxylamine on gold is promoted when the pH is switched from 4 to 9, while the oxidation rate decreases when the pH is higher than 9. In anolyte 4 with an initial pH value of 10.8, two oxidation waves (array a and b in **Figure S9**) were observed in the CV recorded at the Au SECM tip. Since, the p*K*a of hydroxylamine is 5.9, the hydroxylamine is presented at both forms in anolyte 4: NH_2_OH and NH_3_OH^+^. The oxidation peak a and b can be assigned to the oxidation of non-protonated and protonated forms, respectively.^[6]^


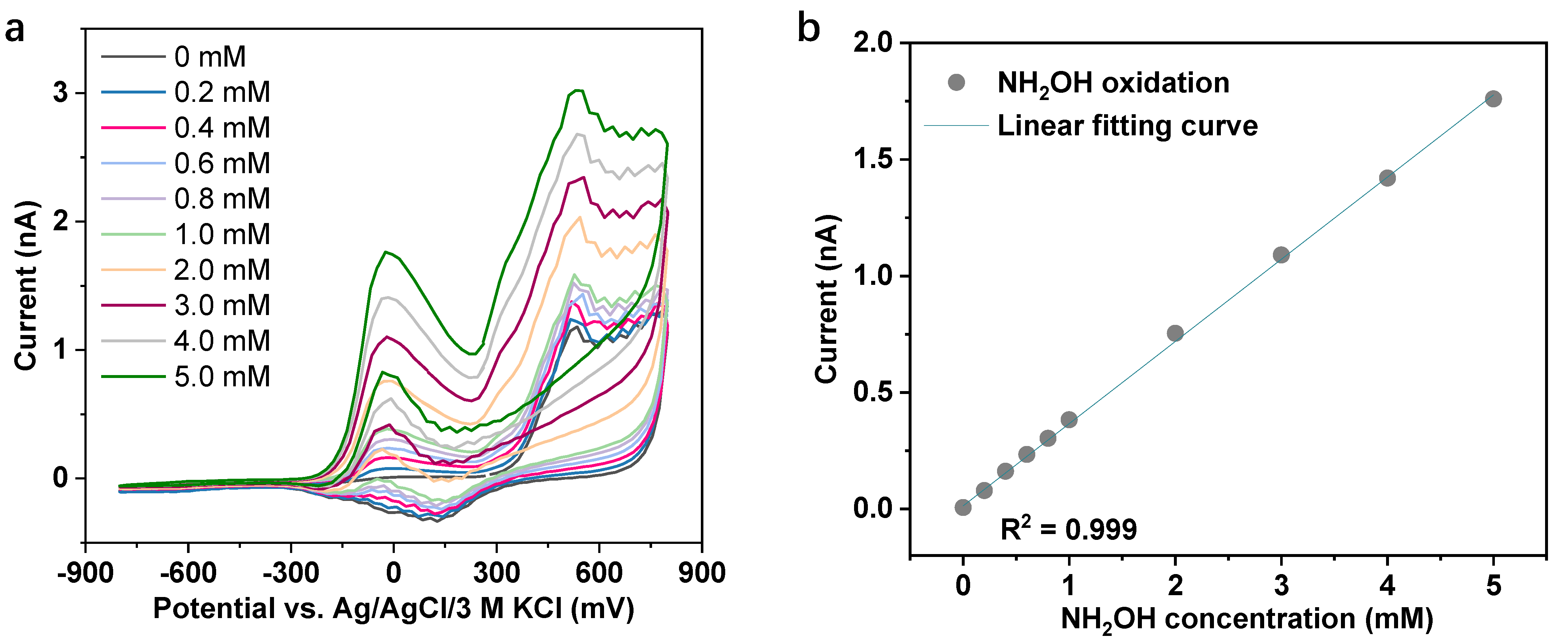


**Figure S10.** (a) CVs at the Au microelectrode in anolyte 4 containing different concentrations of NH_2_OH. (b) Calibration curve for NH_2_OH detection at the Au tip in anolyte 4. CVs in **Figure S10**a demonstrate that NH_2_OH can be oxidized at the Au tip, and a linear correlation was observed by plotting the tip current at -20 mV vs. Ag/AgCl/3 M KCl against the NH_2_OH concentration (**Figure S10**b).


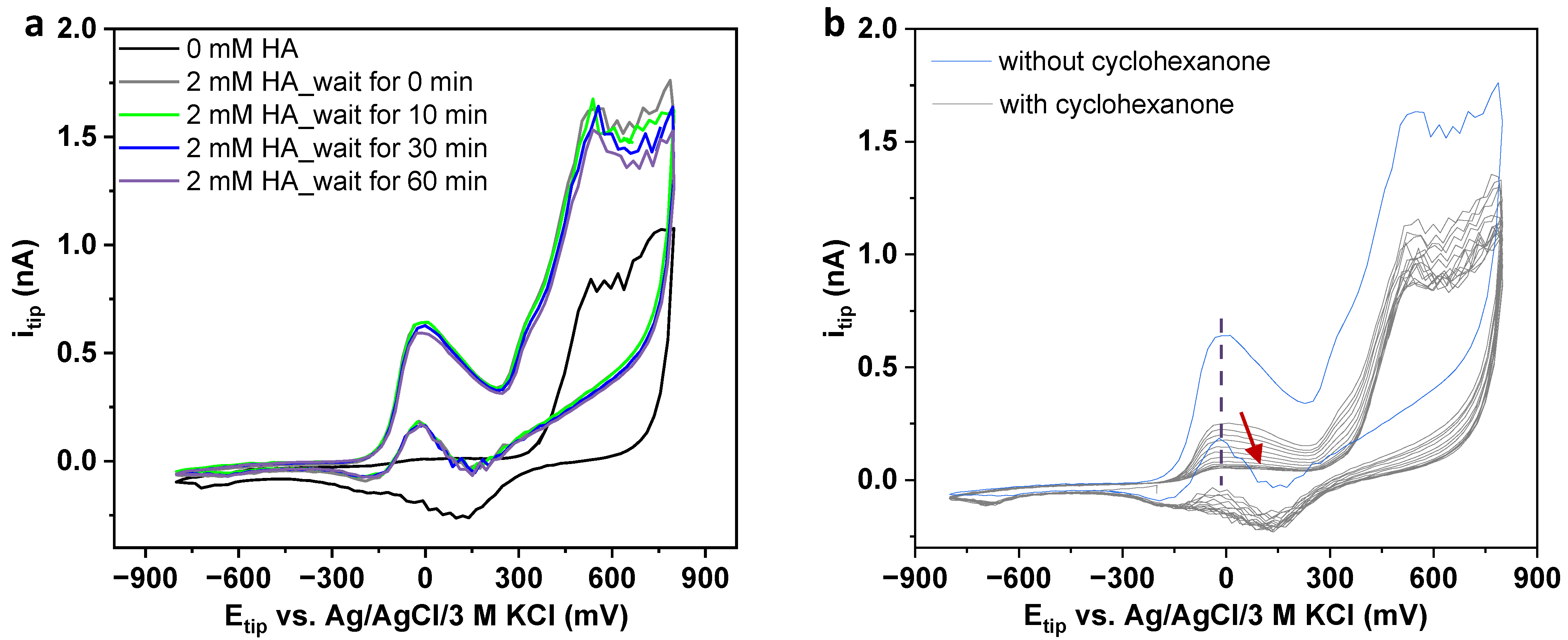


**Figure S11.** (a) Stability test by recording sequential CVs at the Au microelectrode in anolyte 4 containing 0 or 2 mM NH_2_OH after waiting for different times. (b) Stability test performed by recorded sequential CVs at the Au tip with and without cyclohexanone (10 mM). The blue curve is the CV at the Au tip without cyclohexanone. The gray curves are the CVs recorded at the Au tip after adding cyclohexanone (interval time of CVs acquisition of 50 s). The red arrow indicates the CV curves from the first to the last cycle.


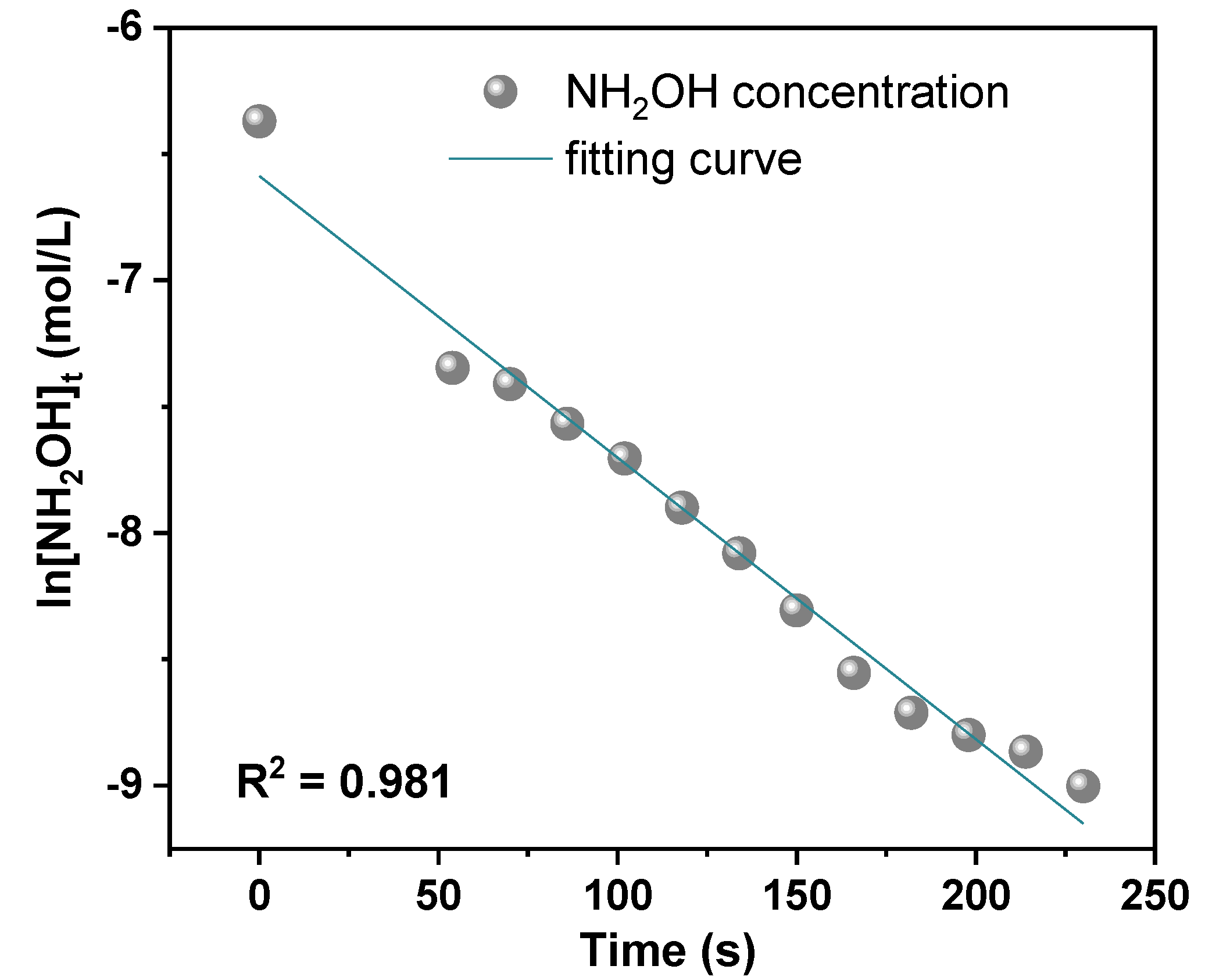


**Figure S12.** ln[NH_2_OH]_t_ plotted against time.

The pseudo-first-order rate constant 𝑘′ was determined to be 0.0111 s⁻¹ based on the linear fit of ln[NH₂OH] versus time (𝑅^2^ = 0.981). Given that the initial concentration of cyclohexanone was 10 mM, the intrinsic rate constant 𝑘 was calculated according to the relation 𝑘′ = 𝑘[cyclohexanone], yielding 𝑘 = 1.11 s^-1^ M^-1^. This value indicates that the reaction proceeds at a moderate rate at alkaline conditions (anolyte 4), consistent with typical nucleophilic addition kinetics in aqueous systems.


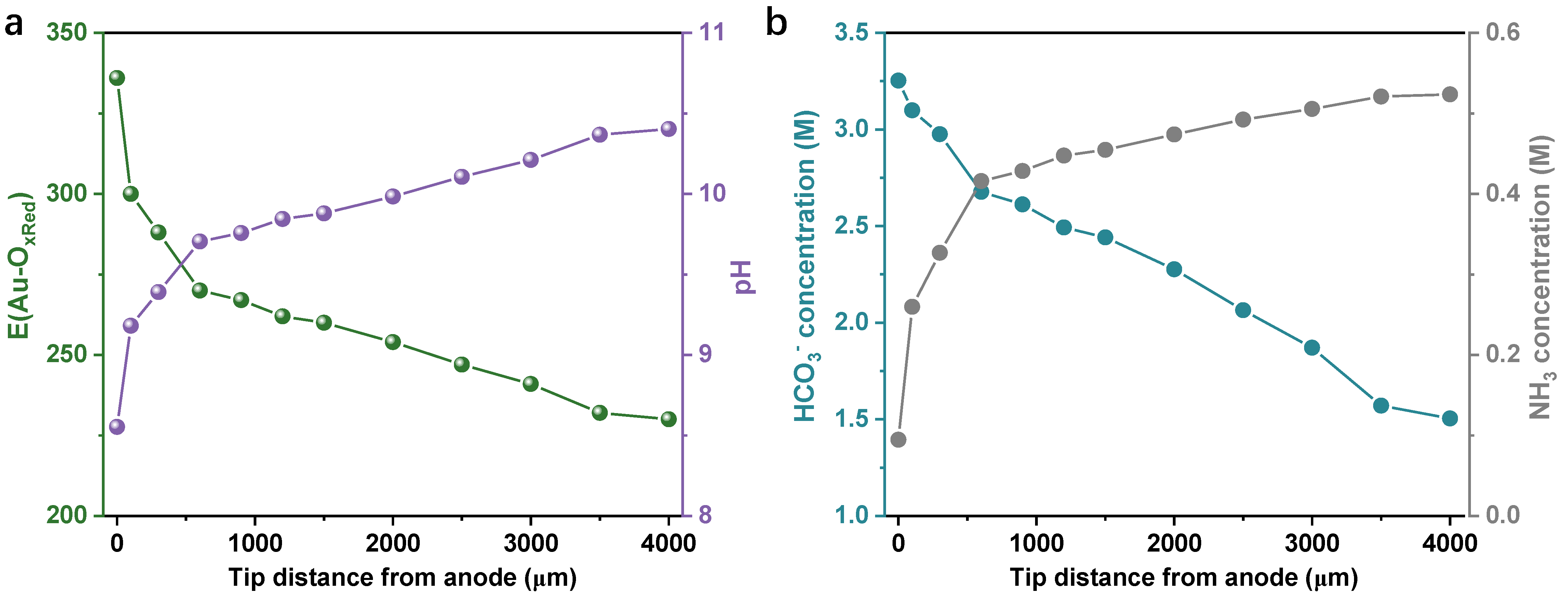


**Figure S13.** (a) E(Au-O_xRed_) values extracted from the Au CVs and the corresponding pH values in the diffusion layer of anolyte 4 as a function of tip-to-anode distance. (b) Calculated concentrations of HCO_3_^-^ and NH_3_ in the diffusion layer as a function of tip-to-anode distance. The position of minimum position was 10 μm away from the approached position. The potential applied to the anode is 2.83 V vs. RHE. All experiments were conducted under natural diffusion conditions with no forced convection.

**
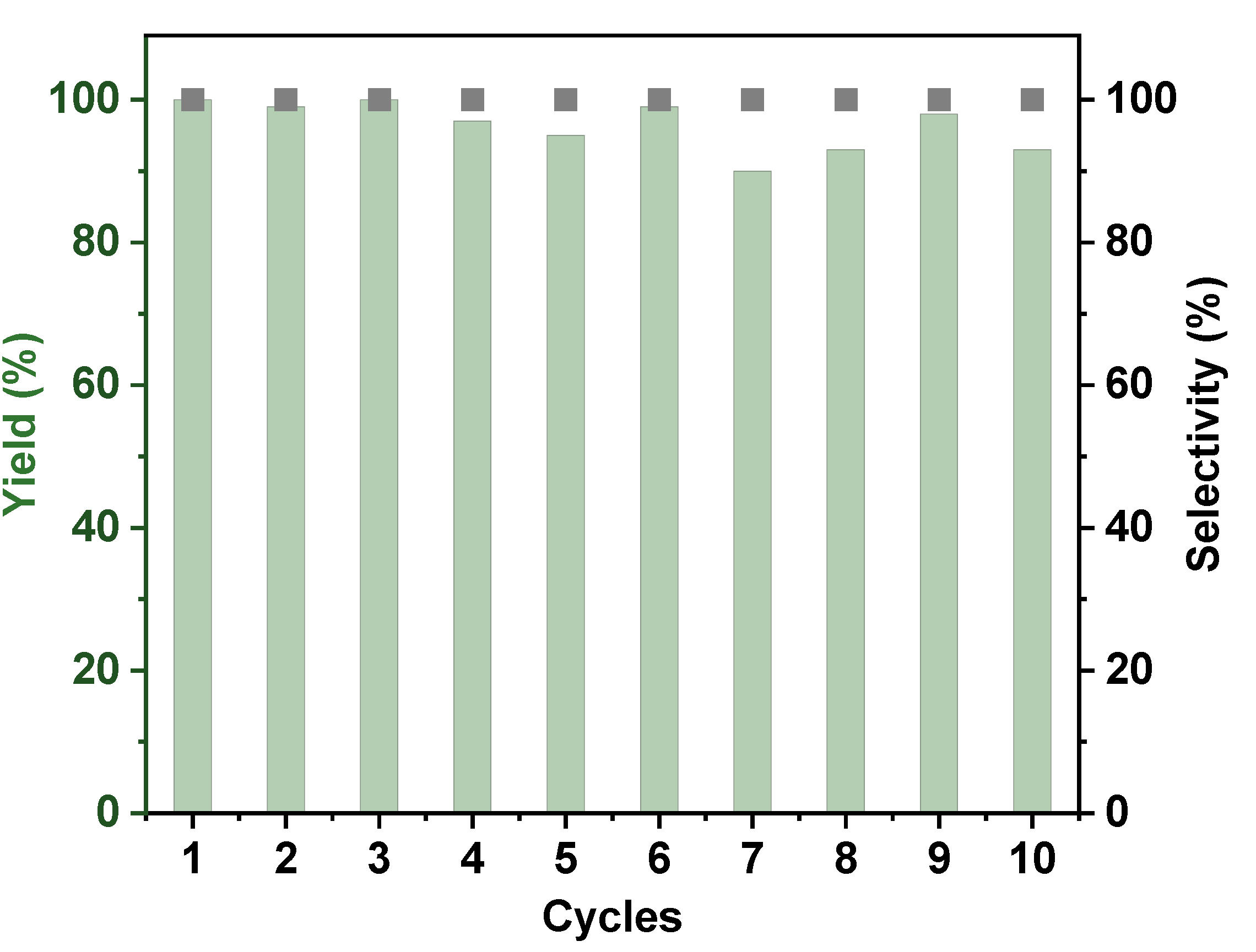
**

**Figure S14.** The performance stability test of anode FTO/Sb_2_WO_6_ for cyclohexanone oxime production.


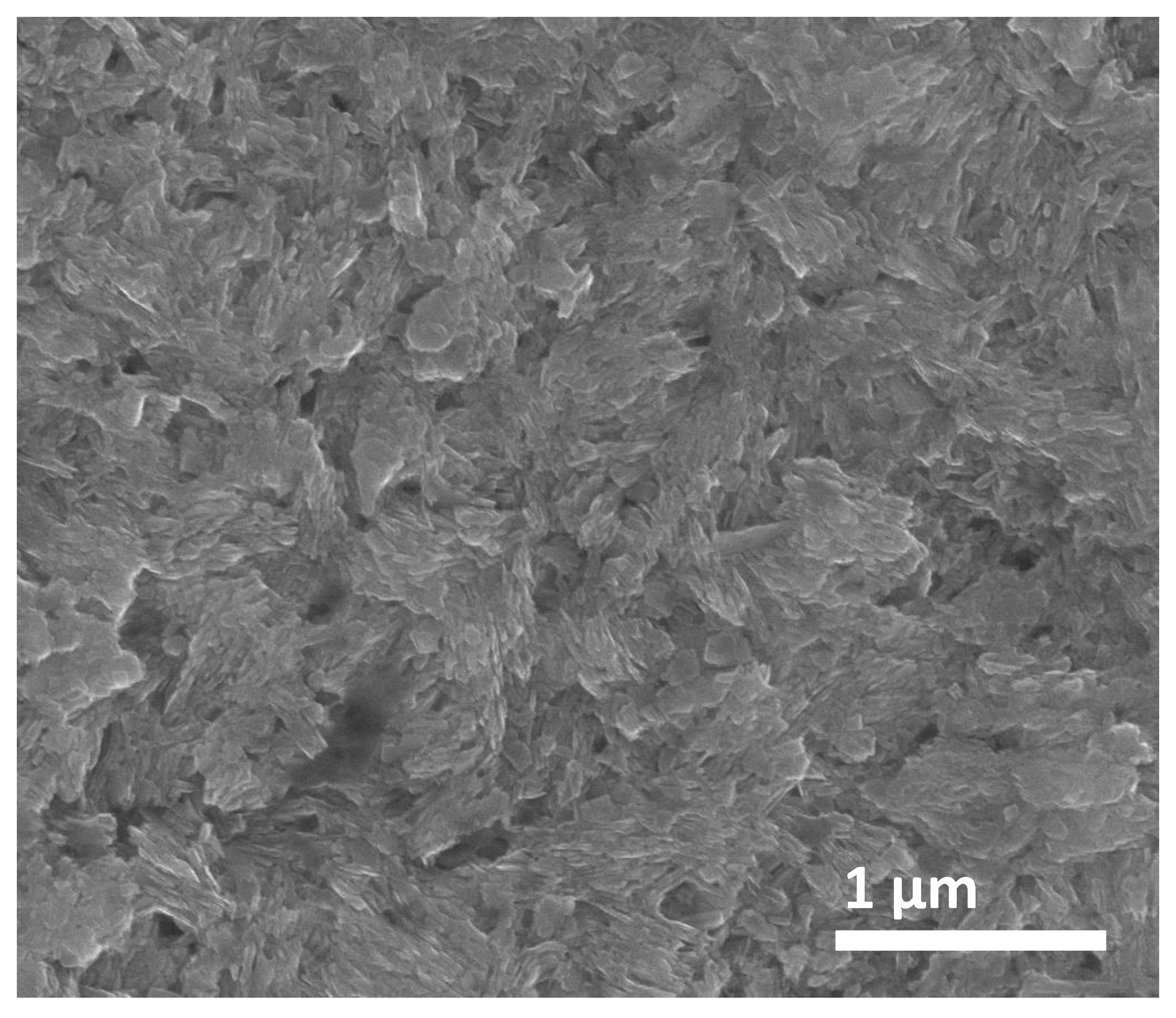


**Figure S15.** SEM image of the FTO/Sb_2_WO_6_ film after 10 cycles of stability test.


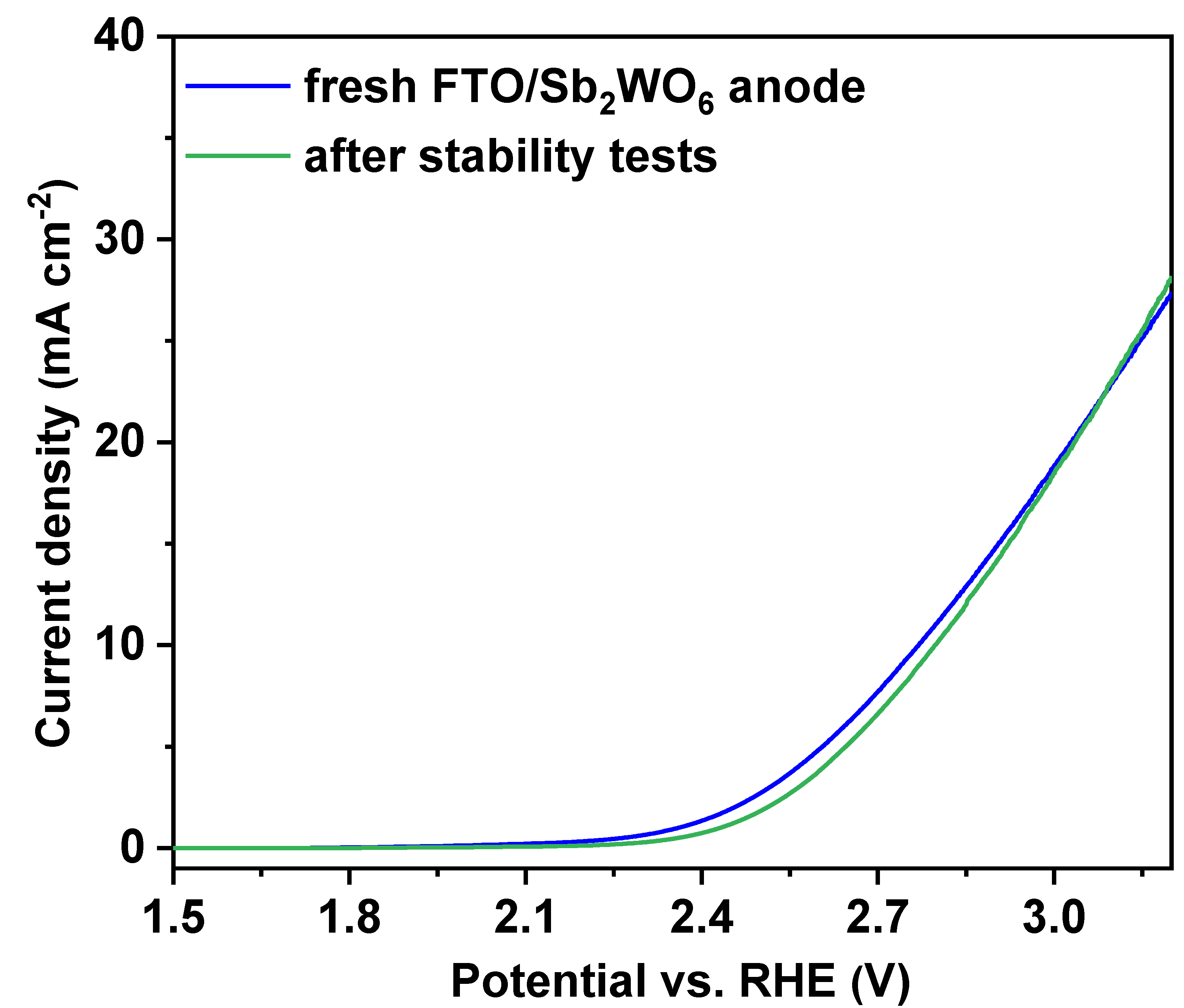


**Figure S16.** LSV curves of the fresh FTO/Sb_2_WO_6_ film and the spent electrode after 10 cycles of the stability test in anolyte 4 with a scan rate of 10 mV s^-1^.


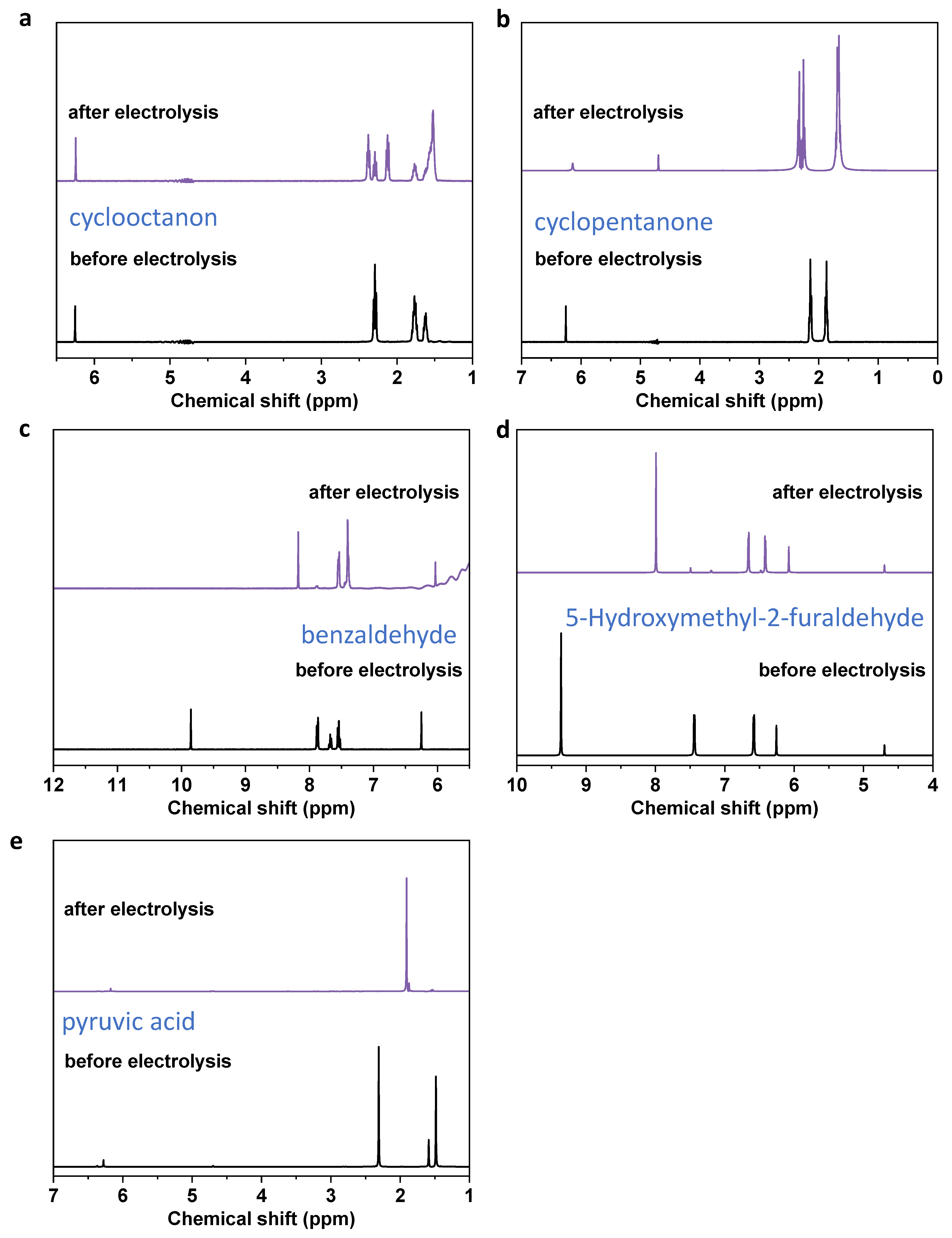


**Figure S17.** The NMR spectra of a variety of other substrates (including cyclooctanone, cyclopentanone, benzaldehyde, hydroxymethylfurfural, pyruvic acid) and the corresponding oximes formed.

**Table S2.** The performance comparison of FTO/Sb_2_WO_6_ and previous reported catalysts for synthesis of cyclohexanone oxime.

| No. | N-source | C-source | catalyst | Potential/  current density | FE/EE | Cyclohexanone oxime yield | Ref |
| --- | --- | --- | --- | --- | --- | --- | --- |
| 1 | NO | cyclohexanone | CP/PTFE | -0.4 V vs. RHE | 47.7%  at -0.3 V vs. RHE | 10.7 mg h^-1^ cm^-2^  at -0.4 V vs. RHE | ^[7]^ |
| 2 | Plasma-assisted air-to-NO_x_ | cyclohexanone | 0.6% Cu/TiO_2_ | -1.8 V vs. Ag/AgCl | 51.4% | 20.1 mg h^-1^ cm^-2^ | ^[8]^ |
| 3 | NO_3_^-^ | cyclohexanone | Fe (110) | -0.3 V vs. RHE | 20.1% | - | ^[9]^ |
| 4 | NO_2_^-^ | cyclohexanone | Cu-S | -0.9 V vs. Ag/AgCl | 26% | 0.165 mmol h^-1^ cm^-2^  (5.4 mg h^-1^ cm^-2^) | ^[10]^ |
| 5 | NO_2_^-^ | cyclohexanone | PdCuAgBi high-entropy alloy | -0.9 V vs. Ag/AgCl | 47.6% | 0.228 mmol h^-1^ cm^-2^ | ^[11]^ |
| 6 | NO_3_^-^ | cyclohexanone | Rutile-TiO_2_ | 30 mA cm^-2^ | 68.2% | 127.3 μmol h^-1^ cm^-2^  (14.4 mg h^-1^ cm^-2^) | ^[12]^ |
| 7 | NO_3_^-^ | cyclohexanone | Zn_93_Cu_7_ | 100 mA cm^-2^  (~ -0.8 V vs. RHE) | 27% | - | ^[13]^ |
| 8 | NO_3_^-^ | cyclohexanone | ZnPc MDE/CNTs | -0.8 V vs. RHE | 53% | 57 mg h^-1^ for NH_2_OH at  -0.85 V vs. RHE | ^[14]^ |
| 9 | NO_3_^-^ | cyclohexanone | Cu_1_MoO_x_/N-doped carbon | -0.5 A cm^-2^ | 94.5% | 3.0 mol h^-1^ g_cat_^-1^) | ^[15]^ |
| 10 | NH_3_ from NH_4_HCO_3_ | cyclohexanone | oxygen-doped  carbon | 0.1 V vs. RHE | 96% | 22.5 mmol h^-1^ g_cat_^-1^ | ^[16]^ |
| 11 | NO_2_^-^ | cyclohexanone | FeBPAbipyH/CNT@CP | -0.4 V vs. RHE | 77.3% | 4.06 mg h^-1^ cm^-2^  (87.00 mg h^-1^ cm^-2^ mg_cat_^-1^) | ^[17]^ |
| 12 | NH_3_ | cyclohexanone | Sb_2_WO_6_ | 2.8 V | 81.3% | 281 μmol h^-1^ cm^-2^  (31.8 mg h^-1^ cm^-2^) | This work |

**References**

[1] X. Shi, S. Siahrostami, G.-L. Li, Y. Zhang, P. Chakthranont, F. Studt, T. F. Jaramillo, X. Zheng, J. K. Nørskov, *Nat. Commun.* **2017**, *8*, 701.

[2] Y. Miyase, Y. Miseki, T. Gunji, K. Sayama, *ChemElectroChem* **2020**, *7*, 2448-2455.

[3] X. Jia, Z. Yu, F. Liu, H. Liu, D. Zhang, E. Campos dos Santos, H. Zheng, Y. Hashimoto, Y. Chen, L. Wei, H. Li, *Ad. Sci.* **2024**, *11*, 2305630.

[4] N. V. Klassen, D. Marchlngton, H. C. E. McGowan, *Anal. Chem.* **1994**, *66*, 2921-2925.

[5] L. Li, R. P. Antony, C. S. Santos, N. Limani, S. Dieckhofer, W. Schuhmann, *Angew. Chem. Int. Ed.* **2024**, *63*, e202406543

[6] P. Kannan, S. A. John, *Anal. Chim. Acta* **2010**, *663*, 158-164.

[7] X. Zhang, H. Jing, S. Chen, B. Liu, L. Yu, J. Xiao, D. Deng, *Chem Catal.* **2022**, *2*, 1807-1818.

[8] S. Jia, X. Tan, L. Wu, X. Ma, L. Zhang, J. Feng, L. Xu, X. Song, Q. Zhu, X. Kang, X. Sun, B. Han, *Chem. Sci.* **2023**, *14*, 13198-13204.

[9] Y. Wu, W. Chen, Y. Jiang, Y. Xu, B. Zhou, L. Xu, C. Xie, M. Yang, M. Qiu, D. Wang, Q. Liu, Q. Liu, S. Wang, Y. Zou, *Angew. Chem. Int. Ed.* **2023**, *62*, e202305491.

[10] Y. Wu, J. Zhao, C. Wang, T. Li, B. H. Zhao, Z. Song, C. Liu, B. Zhang, *Nat. Commun.* **2023**, *14*, 3057.

[11] Y. Sheng, J. Xie, R. Yang, H. Yu, K. Deng, J. Wang, H. Wang, L. Wang, Y. Xu, *Angew. Chem. Int. Ed.* **2024**, *63*, e202410442.

[12] L. Luo, L. Li, L. Xu, Y. Yan, S. Zhang, H. Zhou, Z. Li, M. Shao, X. Duan, *CCS Chem.* **2025**, *7*, 266-278.

[13] J. Sharp, A. Ciotti, H. Andrews, S. R. Udayasurian, M. Garcia-Melchor, T. Li, *ACS Catal.* **2024**, *14*, 3287-3297.

[14] Y. Tang, Z. Jiang, Y. Yuan, L. Xu, C. Jin, B. Chen, Z. Lin, J. Zao, J. Du, X. Zhang, X. Gao, Y. Liang, *Nat. Commun.* **2024**, *15*, 9800.

[15] R. Zhao, Y. Wang, J. Fu, F. Zhang, L. Wen, Y. Zhao, B. Guan, B. Han, Z. Liu, *J. Am. Chem. Soc.* **2024**, *146*, 27956-27963.

[16] Y. Yuan, L. Chen, Z. Wan, K. Shi, X. Ten, H. Xu, P. Wu, J. Shi, *Sci. Adv.* **2023**, *10*, 1755.

[17] C. Zhang, S. L. Meng, Y. N. Jing, C. Wang, X. L. Zhang, H. X. Wang, C. H. Tung, L. Z. Wu, *Angew. Chem. Int. Ed.* **2025**, *64*, e202506546.
